# Study protocol for a randomized controlled trial: Qiliqiangxin in heart failUre: assESsment of reduction in morTality (QUEST)

**DOI:** 10.1186/s12906-020-2821-0

**Published:** 2020-02-05

**Authors:** Wenming Yao, Iokfai Cheang, Shengen Liao, Yanli Zhou, Fang Zhou, Dongjie Xu, Zhenhua Jia, Liping Chang, Haifeng Zhang, Xinli Li

**Affiliations:** 1 Department of Cardiology, the First Affiliated Hospital with Nanjing Medical University, Guangzhou Road 300, Nanjing, 210029 China; 2 National Key Laboratory of Collateral Disease Research and Innovative Chinese Medicine, Shijiazhuang, China; 3 Hebei Yiling Hospital, Key Disciplines of State Administration of TCM for Collateral Disease, Shijiazhuang, China

**Keywords:** Qiliqiangxin (QLQX), Traditional Chinese medicine (TCM), Heart failure, Mortality, Rationale

## Abstract

**Background:**

Qiliqiangxin (QLQX) capsule is a Traditional Chinese Medicine (TCM) that has been approved in China for the treatment of chronic heart failure (CHF). Our previous study showed with a background of standard HF treatment, QLQX capsules further reduced the levels of NT-proBNP and the incidence of composite cardiac events (CCEs) in CHF patients. This study aims to further assess the reduction in mortality when using QLQX compared with placebo for heart failure with reduced ejection fraction (HFrEF) patients.

**Methods:**

This study is a randomized, double-blind, placebo-controlled, parallel-group, multi-center, event-driven clinical study of approximately 3080 patients for a targeted 620 events. Patients must have a diagnosis of heart failure for at least 3 months prior to screening. Patients will be randomized 1:1 to receive the placebo or QLQX in addition to their standard medications of CHF. The primary efficacy outcome event is a composite cardiovascular death and re-hospitalization due to the worsening of heart failure.

**Discussion:**

The QUEST study is a randomized control study of TCM in chronic heart failure. It will determine the place of QLQX as an new treatment approach and provide additional and innovative information regarding TCM - and the specific used of QLQX in HFrEF.

**Trial registration:**

The trial was registered at http://www.chictr.org.cn. ( Registration No.: ChiCTR1900021929); Date: 2019-03-16.

## Background

In the past decade, Cardiovascular disease is one of the major causes of death globally, seriously threatening human life and health [1, 2]. The current annual incidence of heart failure (HF) in the United States and Europe is over one million. And there were an estimated 26 millions patients worldwide suffering from HF [3, 4]. A large-scale registry study in China showed there were over 4.5 million patients with HF (prevalence in Chinese adults aged 35–74 was 0.9%). The mortality rate due to cardiovascular diseases accounted for > 40% of deaths from all-causes in China [2, 5, 6]. The numbers are rapidly increasing due to the aging and expanding population of multiple risk factors for HF, such as aging, hypertension, diabetes mellitus, etc.

In the past 20 years, the concept of drug treatment for heart failure has changed greatly, from the perspective of improving hemodynamics to the point of biological adjustment. Various new treatments and optimized management approaches in heart failure have been suggested. Despite this progress regarding heart failure treatment, the current prevalence and mortality of heart failure remain high. More and more HF patients are turning to complementary and alternative medicine (CAM) to deal with the symptoms and signs of the disease.

The hemodynamic changes caused by early neuroendocrine activation and ventricular remodeling is the basic mechanism in the progressive development of heart failure [7], which is similar from the perspective of traditional Chinese medicine (TCM) [8]. Using the theoretical basis of TCM and modern CAM, Qiliqiangxin (QLQX) was originally designed to treat heart failure.

### Qiliqiangxin

Qiliqiangxin (QLQX) capsule is a specific TCM extract obtained from 11 types of herbs (see “Investigational products” section below). Besides the TCM philosophy, the effect of QLQX capsules might result in preventing phenylephrine-induced cardiac hypertrophy through PPAR-γ and its coactivator PGC-1α [9]. Our previous study showed with background of standard treatment, QLQX further reduced the levels of NT-proB-type Natriuretic Peptide (NT-proBNP), and also demonstrated a superior performance in NYHA heart function, left ventricular ejection fraction (LVEF), 6-min walking distance (6MWD), and for the quality of life [10]. It was approved by the China Food and Drug Administration in 2004 for the treatment of heart failure.

### What has still to be done

QLQX has already showed the effect on attenuating cardiac remodeling in various animal models and the potential to prevent the cardiomyocytes hypertrophy that is characterized in the mechanism of heart failure [11–15]. Our study aims to further explore the efficacy and safety of QLQX capsule compared with placebo in patients with reduced ejection fraction (HFrEF). Herein, we describe the design of “Qiliqiangxin in Heart FailUre: AssESsment of Reduction in MorTality (QUEST)” study.

## Methods/design

### Primary and secondary outcomes

The primary efficacy objective is to demonstrate that QLQX is superior to the placebo in subjects with chronic heart failure (CHF) in addition to conventional heart failure treatment. The primary outcome is the occurrence of the composite endpoint which defined as either cardiovascular (CV) death or re-hospitalization due to the exacerbation of HF.

Secondary outcomes measures include the all-cause mortality, secondary endpoint events (treatment terminated due to worsening heart failure, successful resuscitation after cardiac arrest, malignant arrhythmia, non-fatal stroke), CV death and re-hospitalization due to worsening heart failure in patients with ischemic heart disease, and the level of Serum NT-proBNP.

The assessments of safety and tolerability were based on spontaneous reports of adverse events, vital signs, ECG, and laboratory measurements (complete blood count, routine urinalysis, serum biochemistry, etc.). All endpoint events will be reviewed and determined by the Clinical Event Adjudication Committee (Additional file 1).

### Design and patient population

This study is a randomized, double-blind, placebo-controlled, parallel-group, event-driven, multicenter clinical study.

The study will be event-driven and it is expected that 3080 patients in over 100 centers (mainland China, HongKong, Macau) will be enrolled. All eligible sites are hospital based and are tertiary referral hospitals. Hospitals must have sufficient resources, prior trial experience or a patient population meeting study the selection criteria to allow for adequate recruitment and be able to provide standard heart failure treatment.

All randomized patients will remain in the study (whether taking the study drug or not) until the number of primary endpoint events reaches the predicted value (620 cases), or the study terminates early when it meets the pre-defined efficacy or safety criteria for early termination assessed by the committees (Additional file 1).

The QUEST study will enroll adult (≥18 years old) patients, who are clinically stable up to 30 days after a symptomatic event. At the screening visit, patients from both inpatient or outpatient can be assessed according to the inclusion/exclusion criteria (Table 1). Patients need to fulfil all the the inclusion criteria including the LVEF, NT-proBNP, serum sodium, serum potassium, and estimated glomerular filtration rate (eGFR), received treatment, etc., and not meet any exclusion criterion.

**Table 1:** Inclusion and exclusion criteria

Inclusion criteria:	
1) Signed informed consent (Additional file 3.);	
2) Aged ≥18 years at the time of consent;	
3) Established documented diagnosis of heart failure for at least 3 months ago according to “Chinese Heart Failure Diagnosis and Treatment Guideline” issued by the Chinese Medical Association Cardiovascular Branch.	
4) Left ventricular ejection fraction (LVEF) ≤40% (echocardiogram, radionuclide, ventriculogram, contrast angiography or cardiac MRI);	
5) NYHA cardiac functional grading II to III, with stable clinical symptoms; including those diagnosed as grade IV within 2 weeks before enrollment;	
6) Serum NT-proBNP ≥450 pg/ml;	
7) Those who have received standardized baseline treatment regimens without doses adjusted and given intravenously for at least 2 weeks prior to enrollment;	
8) standardized drug treatment includes: angiotensin-converting enzyme inhibitor (ACEI) or angiotensin receptor blocker (ARB) or angiotensin receptor neprilysin inhibitor (ARNI), beta blocker, and aldosterone receptor antagonist (the optimal therapeutic dose should be achieved, except for contraindications or intolerance).	
Exclusion criteria:	
1) Patients should not enter the study if any of the following exclusion criteria are fulfilled	
2) Heart failure caused by valvular disease, congenital heart disease, pericardial disease, arrhythmia or non-cardiaogenic disease, or caused by vital organ failure (such as renal, hepatic failure, etc.); and right heart failure caused by pulmonary or other definite causes; and acute heart failure;	
3) Coronary revascularization (percutaneous coronary intervention [PCI] or coronary artery bypass grafting [CABG]) or cardiac synchronization therapy planned to undergo after randomization, or had received cardiac resynchronization therapy prior to enrolment;	
4) Any condition outside the CV diseases such as but not limited to malignant tumor, severe mental illness, hematopoietic diseases, neuroendocrine system disease, liver transaminase and alkaline phosphatase ≥3 x upper limit of normal (ULN), abnormal renal function serum creatinine > 2 mg/dl (176.82 umol/L), potassium > 5.5 mmol/L;	
5) Patient with left ventricular outflow tract obstruction, myocarditis, aortic aneurysm, aortic dissection, or obvious hemodynamic changes caused by unrepaired valve;	
6) Cardiogenic shock, uncontrollable malignant arrhythmia, sinus or atrioventricular block at second degree type II or above without pacemaker treatment, progressive unstable angina pectoris or acute myocardial infarction;	
7) uncontrolled hypertension systolic blood pressure (SBP) ≥ 180 mmHg and/or diastolic blood pressure (DBP) ≥ 110 mmHg; or SBP < 90 mmHg and/or DBP < 60 mmHg;	
8) Participation in another clinical study with an IP during the last month prior to enrolment;	
9) Women of child-bearing potential (i.e., those who are not chemically or surgically sterilized or who are not post-menopausal) who are not willing to use a medically accepted method of contraception that is considered reliable in the judgment of the investigator, from the time of signing the informed consent throughout the study and 4 weeks thereafter, OR women who have a positive pregnancy test at enrolment or randomisation OR women who are breast-feeding;	
10) Allergic constitution; known to be allergic to research drug;	
11) Inability of the patient, in the opinion of the investigator, to understand and/or comply with study medications, procedures or any conditions may render the patient unable to complete the study.	

**Fig. 1:**
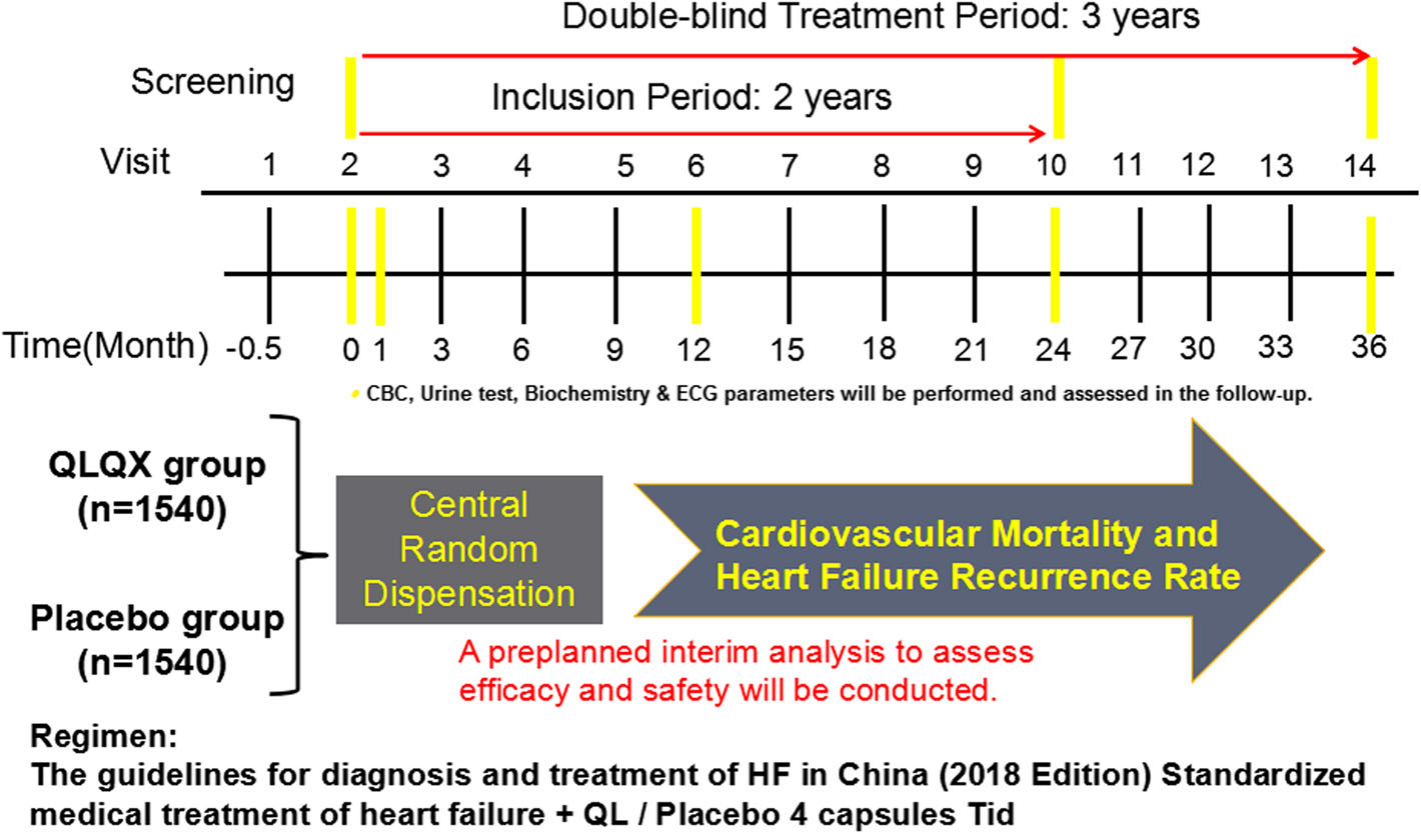
Study flowchart

The entire study will last for about 36 months, and the recruitment period are expected to be 24 months. Participants will be monitored and assessed by the investigators at each study visit. Termination of the follow-up period will be 12 months after the last eligible enrolled case. Therefore, the shortest follow-up period will be at least 12 months and the average follow-up time will be about 24 months. Patients should visit the hospital for efficacy and safety assessments during the 1st, 3rd, 6th, 9th, and 12th months after the random grouping until the study finishes. Study flowchart is shown in Fig. 1 and the detailed study design is shown in Additional file 2.

**Fig. 2:**
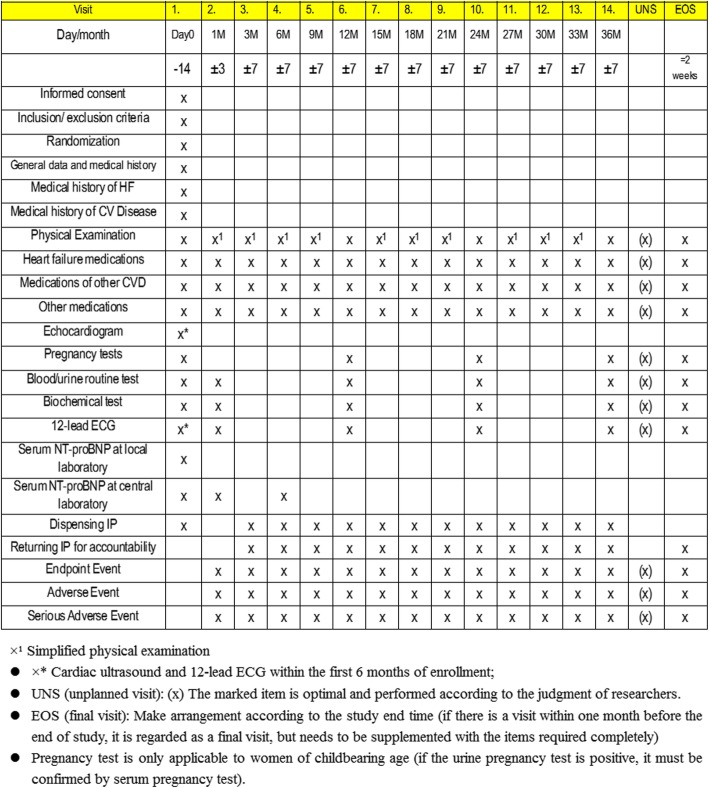
Planned visits and parameters

Corresponding parameters shown in Fig. 2 including lab and ECG results will be recorded or assessed at each follow-up by the responsible physician on each site. A Data Safety Monitoring Committee (DSMC) will oversee safety in QUEST. The assessment of safety will be based primarily on the frequency of adverse events, SAEs, and laboratory abnormalities. The study plans to perform two interim efficacy analyses after collecting 1/2 and 2/3 primary endpoints (i.e., approximately 310 and 414 patients, respectively) to identify early in the course of the study any potential safety or tolerability issues and assess whether a valid conclusion has been reached allowing the committee to terminate the study early. The modification of research program should formulate modification description and be carried out after submitting to the Ethics Committee for approval.

This study used Epidata software to collect research data. Throughout the study, data safety monitoring committee will execute the audit and monitoring of research protocol adherence with the assistance of trained clinical research coordinator and clinical research auditor.

### Sample size and statistical methods

It is expected that 3080 patients in over 100 centers (1540 patients per group, 1:1 division into the study group and the control group) will be enrolled and followed up for at least for 12 months. Sample size was estimated referring to the PARADIGM-HF study [16], cardiovascular death or hospitalization rate for heart failure in the median follow-up of 27 months was 21.8% in the LCZ696 group and 26.5% in the Enalapril group. We estimate the incidence of cardiovascular death and hospitalization for heart failure is 25% in all patients receiving basic treatment +placebo group within 36 months of follow-up and 20% with those receiving basic treatment + QLQX Capsule group. Sample size is estimated by PASS13 software.

The random distribution ratio is 1:1 between the study group and the control group. Considering the consumption of type I error in the interim analysis, α is adjusted to unilateral 0.02314. The sample size is the number of cases with composite endpoints events. It is expected that 620 composite endpoint events need be observed to provide 80% power of test (β = 0.2), and the 20% risk can be reduced in the study group by the log-rank test.

Statistical specialists at Peking University Clinical Research Institute adopts SAS®9.4 software (or higher version) package to generate random numbers using the block randomization method according to the ratio of 1:1 between study group and control group. The study drug (QLQX or placebo capsules) will be packaging according to this random number by a person unrelated to the study.

A randomization and trail supply management system (RTSM) is used in the study, and statistical professionals will provide a random numbered list to the RTSM. The patient is then assigned a random number by the RTSM. Unblinding will be available in case of adverse events.

After completing baseline assessment, random numbers are assigned by RTSM during baseline visits. After that, the drug number is obtained through the RTSM according to the interview plan, and the serial number of drug assigned each time is different, but the drugs are the same. Before randomization of the patient, the researcher must first log into the RTSM and provide the according information (e.g. the subject’s date of birth, gender, etc.).

Two interim analyses during and full analysis after the study will be performed by independent statisticians from Peking University Clinical Research Institute who will not be involved in the trial conduct. Demographic characteristics, efficacy analysis, compliance analysis, and safety analysis will be performed in these time points. *P* value less than or equal to 0.05 (two-sided test) is considered with statistical difference (unless specified otherwise). Our descriptive statistics will include means and SDs, medians and interquartile ranges for continuous variables, and the number and proportions for categorical variables as appropriate. We will compare the two intervention groups regarding their characteristics with paired t-test, Wilcoxon rank sum test, chi-square test, precise probability method, or Cochran-Mantel-Haenszel test based on the type of variables.

### Investigational products (IP)

QLQX and the matching placebo capsules have identical characters as shown in Table 2. IPs will be packed in identical package and label. Eligible patients were randomly assigned to 2 groups that received either QLQX or a placebo (in a 1:1 ratio; double blinded; provided as identical in size and shape) in addition to their standard HF treatment following the guidelines for diagnosis and treatment of HF in China 2018 [17] or local guideline. Participants should not have used any TCM or herbs having similar contents to the IP, such as Danshen and Tongxinluo Capsules. The dosage used in this study was 4 capsules of QLQX or placebo 3 times daily. Patients were recommended to administrate the IPs 30 min after meal, and should also be separated by a minimum 30 min interval with other medications.

**Table 2:** Characteristics of the Investigational Product

Study drug: Qiliqiangxin Capsule (芪苈强心胶囊)	
-Ingredients: Astragalus, Ginseng, Monkshood, Danshen, Pepperweed Seed, Rhizoma Alismatis, Radix Polygonati Officinalis, Cassia Twig, Red Flower, Cortex Periplocae, Tangerine Peel	
-Property: Capsule; the contents are brown to black brown granules; bitter in taste;	
-Specification: 0.3 g/granule	
-Bach number: GYZZ Z20040141	
-Manufacturer: Shijiazhuang Yiling Pharmaceutical Co., Ltd.	
Placebo: Qiliqiangxin Matching Placebo	
-With identical color, specification, packaging, property of contents and other features with Qiliqiangxin Capsule	

At any time after randomization, patients are free to discontinue for any reason. The reason for the withdrawal should be acquired and recorded in the case report form. If the patient has an intolerable adverse event, which is relevant to the study drug according to the judgment of researcher, the patient should terminate treatment with the study drug.

## Discussion

Recent years, HF treatment strategy based on dual RAAS blockade and natriuretic peptide augmentation have been explored. The prognosis for heart failure has improved based on the optimal use of neurohormonal antagonists such as angiotensin-converting enzyme inhibitors, beta-blockers, aldosterone antagonists, and neprilysin inhibitors [18]. Despite advances in management and treatment of chronic heart failure with reduced ejection fraction (HFrEF), HF continues to be a major cause of mortality, initial and recurrent hospitalizations, and sub-optimal quality of life. On the other hand, myocardial remodeling is the basic mechanism of heart failure, including pathological cardiomyocyte hypertrophy with re-expression of embryonic genes, cardiomyocyte apoptosis and necrosis, and excessive deposition or degradation of myocardial extracellular matrix [19]. Researches regarding these mechanism in HF treatment continues to be undertaken.

Qiliqiangxin (QLQX) is a widely used TCM in CHF treatment in China. With the assistance of translational medical science, its efficacy against cardiac hypertrophy and remodeling has been demonstrated in various studies. Results showed the effects of QLQX in multiple mechanism pathways in treating CHF, such as by regulating the balance of proinflammatory and anti-inflammatory cytokines [20], down-regulating the cardiac chymase signaling pathway and chymase-mediated angiotensin II production [21], inhibiting the myocardial inflammation and cardiomyocyte death to promoted cardiomyocyte proliferation [22].

In our previous multi-centers, randomized, double-blind, parallel-group, placebo-controlled study [10], a total 512 patients were enrolled to explored the effects of QLQX. Results showed on a background of standard treatment, QLQX capsules further reduced the levels of NT-proBNP. Furthermore, fewer deaths and re-admissions for heart failure were observed in the Qiliqiangxin group. Although the mortality differences between groups were not significant, these suggested QLQX in combination therapy for CHF could improve the outcome of patients with CHF. Therefore, to explore the efficacy and safety of QLQX in HFrEF patients, this study include a larger cohort and a longer followup period.

In QUEST study, a longer-term follow up will lead to a better understanding of the comparative benefit regarding the effect of QLQX in CHF and will answer the question of our last relatively short-term randomized controlled study.

The cohort designs is similar PARADIGM-HF. However, QUEST study has the threshold of NT-proBNP ≥450 pg/mL. Although the clinical value of BNP and NT-proBNP were well-established and are the most widely used biomarkers in differential diagnosis, risk stratification and prognostic evaluation of heart failure; nowadays with the increasing number of patients in the regimen of angiotensin receptor-neprilysin inhibitor (ARNi), BNP level can be affected significantly [23] and therefore were not included.

Also, as mentioned above, the mortality differences between groups were not significant in our previous relatively short-term study. A larger population and the basis of CV mortality to detect a clinically meaningful reduction in the primary composite outcome are estimated by the statistic specialist. The primary outcome of CV death or heart failure hospitalization was chosen to further address questions regarding the hard endpoint events in HFrEF patients.

Our study officially started in May, 2019 and is now ongoing, collecting patients and data. Regarding TCM in treatment of heart failure, the QUEST study is an event-driven randomized control study designed to provide additional information on TCM and further assess the effectiveness of Qiliqiangxin capsules in HFrEF patients.

## Supplementary information

**Additional file 1.** List of the QUEST Committees and Investigators.

**Additional file 2.** 2019–05 QUEST-EN (only for review).

**Additional file 3.** Informed consent form.

## Data Availability

Not applicable.

## Abbreviations

6MWD: 6-min Walking Distance
ARNi: Angiotensin Receptor-Neprilysin Inhibitor
CCE: Composite Cardiac Event
CHF: Chronic Heart Failure
CV: Cardiovascular
DSMC: Data Safety Monitoring Committee
eGFR: Estimated Glomerular Filtration Rate
HF: Heart Failure
HFrEF: Heart Failure with Reduced Ejection Fraction
IP: Investigational Products
LVEF: Left Ventricular Ejection Fraction
NT-proBNP: NT-proB-type Natriuretic Peptide
NYHA: New York Heart Association
QLQX: Qiliqiangxin
TCM: Traditional Chinese Medicine
