# Supplementary material for: Study protocol for a randomized controlled trial: Qiliqiangxin in heart failUre: assESsment of reduction in morTality (QUEST)
Source: BMC Complement Med Ther. 2020 Feb 5;20:38. doi: 10.1186/s12906-020-2821-0 (PMC7076750; doi:10.1186/s12906-020-2821-0)
Supplement: Supplementary file 1 — Additional file 1. List of the QUEST Committees and Investigators. [file 12906_2020_2821_MOESM1_ESM.doc]

**Additional file 1 Complete list of the QUEST Committees and Investigators**

Academic Counselling Committee

China: Academician GAO Runlin, Academician ZHANG Boli, Academician ZHANG Yun, Academician GE Junbo, Academician HAN Yaling, Professor HUANG Chongxin

USA: Professor Anthony Rosenzweig

Data Safety Monitoring Committee

Professor CHEN Feng, Professor ZHU Jun, Professor TANG Qizhu, Professor MA Changsheng, Professor CAI Naisheng

Clinical Event Adjudication Committee

Professor ZHANG Shuyang, Professor YANG Xinchun; Professor SONG Lei, Professor CAI Naisheng, Professor CHEN Hong, Professor FAN Weihu, Professor ZHU Wenlin
