# Supplementary material for: Study protocol for a randomized controlled trial: Qiliqiangxin in heart failUre: assESsment of reduction in morTality (QUEST)
Source: BMC Complement Med Ther. 2020 Feb 5;20:38. doi: 10.1186/s12906-020-2821-0 (PMC7076750; doi:10.1186/s12906-020-2821-0)
Supplement: Supplementary file 2 — Additional file 2. 2019–05 QUEST-EN (only for review). [file 12906_2020_2821_MOESM2_ESM.doc]

**The content of this study protocol is confidential and is intended for use by clinical researchers. The material is the property of the sponsor or its sub-units and may not be copied or distributed to persons unrelated to the study.**

Qiliqiangxin in Heart FailUre:

AssESsment of Reduction in MorTality

**(QUEST STUDY)**

**Study Protocol**

**Protocol No.: SP-YFC-05-QUEST**

**Version No.：V1.0**

**Version date: July 10, 2018**

**Registration No. at American ClinicalTrials.gov ************

**Principal investigator: Professor LI Xinli**

**The First Affiliated Hospital of Nanjing Medical University**

**Duration of study: August 2018 to August 2021**

**RESPONSIBLE PARTIES**

**Principal Investigator of the protocol**

| **Research unit** | **Principal investigator** |
| --- | --- |
| The First Affiliated Hospital of Nanjing Medical University | LI Xinli Signature:  Date: |

**Statistical Analysis**

| **Unit** | **Contact** |
| --- | --- |
| Peking University Clinical Research Institute | YAO Chen |

**Drug Manufacturer**

| **Unit** | **Contact** | **Tel** | **Email** |
| --- | --- | --- | --- |
| Shijiazhuang Yiling Pharmaceutical Co., Ltd | HAN Shuolong | 13582167153 | hanshuolong@yiling.cn |

**Academic Counselling Committee**

**China：Academician GAO Runlin, Academician ZHANG Boli, Academician ZHANG Yun, Academician GE Junbo, Academician HAN Yaling, Professor HUANG Chongxin**

**USA：Professor Anthony Rosenzweig**

**Data Safety Monitoring Committee**

**Professor CHEN Feng, Professor ZHU Jun, Professor TANG Qizhu,**

**Professor MA Changsheng, Professor CAI Naisheng**

**Clinical Event Adjudication Committee**

**Professor ZHANG Shuyang, Professor YANG Xinchun; Professor SONG Lei, Professor CAI Naisheng, Professor CHEN Hong, Professor FAN Weihu, Professor ZHU Wenling**

**Abbreviations**

| ACEI | Angiotensin converting enzyme inhibitor |
| --- | --- |
| ADR | Suspected adverse drug reaction |
| AEs | Adverse events |
| AHA | American Heart Association |
| AMI | Acute myocardial infarction |
| ARB | Angiotensin receptor blocker |
| ARNI | Angiotensin receptor neprilysin inhibitor |
| BNP | B-type natriuretic peptide |
| CEA | Clinical Event Adjudication |
| CGRP | Calcitonin gene-related peptide |
| CHF | Chronic heart failure |
| CRA | Clinical research auditor |
| CRC | Clinical research coordinator |
| CRF | Case report form |
| CV | Cardiovascular |
| DSMC | Data and Safety Monitoring Committee |
| DVP | Data verification plan |
| ECG | Electrocardiogram |
| EF | Ejection fraction |
| EOS | Final visit |
| ESC | European Society of Cardiology |
| ET | Endothelin |
| FAS | Full analysis set |
| HF | Heart failure |
| IP | Investigational Product |
| LVOT | Left ventricular outflow |
| MedDRA | ICH International Medical Dictionary |
| MI | Myocardial infarction |
| NO | Nitric oxide |
| NT-proBNP | N-terminal pro brain natriuretic peptide |
| NYHA | New York Heart Association |
| PPS | Per protocol set |
| RAAS | Renin-angiotensin-aldosterone system |
| SAE | Serious adverse event |
| SNS | Sympathetic nervous system |
| SAS | Safety analysis set |
| TVI | Time velocity integral |
| UNS | Unplanned visit |

**PROTOCOL SYNOPSIS**

| **Objectives** | Evidence-based medical research methods are used to explore the clinical efficacy and safety of long-term use of Qiliqiangxin capsule with cardiovascular mortality and incidence of re-hospitalization for recurrence of heart failure as main study endpoints, in order to determine its curative effects and target group, and provide high quality clinical evidence for optimizing clinical drug regimen. | |
| --- | --- | --- |
| **Study design** | Randomized, double-blind, placebo-controlled, multicenter clinical trial | |
| **Target patient population** | **Inclusion criteria:**   1. Provision of signed informed consent prior to any study specific procedures; 2. Male or female, aged ≥18 years at the time of consent; 3. Established documented diagnosis of heart failure for at least three months ago according to “Chinese Heart Failure Diagnosis and Treatment Guideline” issued by the Chinese Medical Association Cardiovascular Branch. 4. Left ventricular ejection fraction (LVEF) ≤40% (echocardiogram, radionuclide, ventriculogram, contrast angiography or cardiac MRI ); 5. NYHA cardiac functional grading II to III, with stable clinical symptoms; including those diagnosed as grade IV within 2 weeks before enrollment; 6. Serum NT-proBNP ≥ 450pg/ml; 7. Those who have received standardized baseline treatment regimens without doses adjusted and given intravenously for at least two weeks prior to enrollment;  - standardized drug treatment includes: angiotensin-converting enzyme inhibitor (ACEI) or angiotensin receptor blocker (ARB) or angiotensin receptor neprilysin inhibitor (ARNI), beta blocker, and aldosterone receptor antagonist (the optimal therapeutic dose should be achieved, except for contraindications or intolerance);   **Exclusion criteria:**  Patients should not enter the study if any of the following exclusion criteria are fulfilled   1. Heart failure caused by valvular disease, congenital heart disease, pericardial disease, arrhythmia or non-cardiaogenic disease, or caused by vital organ failure (such as renal, hepatic failure, etc.); and right heart failure caused by pulmonary or other definite causes; and acute heart failure; 2. Coronary revascularization (percutaneous coronary intervention [PCI] or coronary artery bypass grafting [CABG]) or cardiac synchronization therapy planned to undergo after randomization, or had received cardiac resynchronization therapy prior to enrolment; 3. Any condition outside the CV diseases such as but not limited to malignant tumor, severe mental illness, hematopoietic diseases, neuroendocrine system disease, liver transaminase and alkaline phosphatase ≥ 3 x upper limit of normal (ULN), abnormal renal function serum creatinine > 2 mg/dl (176.82 umol/L), potassium >5.5mmol/L; 4. Patient with left ventricular outflow tract obstruction, myocarditis, aortic aneurysm, aortic dissection, or obvious hemodynamic changes caused by unrepaired valve; 5. Cardiogenic shock, uncontrollable malignant arrhythmia, sinus or atrioventricular block at second degree type II or above without pacemaker treatment, progressive unstable angina pectoris or acute myocardial infarction; 6. uncontrolled hypertension systolic blood pressure (SBP) ≥ 180mmHg and/or diastolic blood pressure (DBP) ≥ 110mmHg; or SBP < 90mmHg and/or DBP <60mmHg; 7. Participation in another clinical study with an IP during the last month prior to enrolment; 8. Women of child-bearing potential (i.e., those who are not chemically or surgically sterilized or who are not post-menopausal) who are not willing to use a medically accepted method of contraception that is considered reliable in the judgment of the investigator, from the time of signing the informed consent throughout the study and 4 weeks thereafter, OR women who have a positive pregnancy test at enrolment or randomization OR women who are breast-feeding;   10）Allergic constitution; known to be allergic to research drug;  11）Inability of the patient, in the opinion of the investigator, to understand and/or comply with study medications, procedures or any conditions may render the patient unable to complete the study. | |
| **Outcome Measures** | Primary endpoints | The composite endpoint events consisting of cardiovascular death and re-hospitalization due to the worsening of heart failure |
|  | Secondary endpoints | 1. All-cause mortality 2. Secondary endpoint events (given up treatment due to worsening heart failure, successful resuscitation after cardiac arrest, malignant arrhythmia, non-fatal stroke) 3. The incidence of cardiovascular death and re-hospitalization due to worsening heart failure in patients with ischemic heart disease 4. Serum NT-proBNP decrease rate |
|  | Safety objective and measure | To evaluate the safety and tolerability of QLQX in this patient population;  Changes in clinical chemistry/haematology parameters, ECG, physical examination, any forms of adverse events (AEs) |
| **Statistical methods** | The primary objective of the study is to determine the superiority of QLQX versus placebo in reducing the incidence of the primary composite endpoint. The random distribution ratio is 1:1 between study group and control group. The sample size is in estimating the incidence of composite endpoint events for 25% during the follow-up period of 36 months. In total 3080 cases were expected to enroll (1540 patients per group) with 620 composite endpoint events should be observed. | |
| **Statistical unit** | Peking University Clinical Research Institute | |
| **Dosage and mode of administration** | Study group: Standardized treatment of chronic heart failure + Qiliqiangxin Capsules, 4 capsules/time, 3 times/day, take orally  Control group: Standardized treatment of chronic heart failure + Placebo Capsules, 4 capsules/time, 3 times/day, take orally | |
| **Duration of treatment** | The patient was enrolled with observation event as the index. The planned enrollment time was 2 years and the drug was taken for at least a year. | |
| **Expected progress** | August 2018 to August 2021 | |

**Contents**

[1. Introduction 8](#__RefHeading___Toc4282)

[1.1 Study Topic 8](#__RefHeading___Toc15772)

[1.2 Study Purpose 8](#__RefHeading___Toc584)

[1.3 Study Background 8](#__RefHeading___Toc20151)

[2. Study design 11](#__RefHeading___Toc25796)

[2.1. Rationale for study design 11](#__RefHeading___Toc4231)

[2.2. Control group and Sample size 11](#__RefHeading___Toc25518)

[2.3. Rationale for study population 11](#__RefHeading___Toc18005)

[2.3.1 . Inclusion criteria 12](#__RefHeading___Toc11194)

[2.3.2 . Exclusion criteria 12](#__RefHeading___Toc11887)

[2.4. Patient enrolment and Follow-up visit 13](#__RefHeading___Toc15577)

[2.5. Discontinuation of investigational product (IP) 13](#__RefHeading___Toc107)

[2.6 Withdrawal 13](#__RefHeading___Toc4590)

[2.7. Discontinuation of the study 14](#__RefHeading___Toc3755)

[3. Treatment 14](#__RefHeading___Toc29396)

[3.1 Investigational products (IP) 14](#__RefHeading___Toc11436)

[3.1.1 Package and label 14](#__RefHeading___Toc11223)

[3.1.2 Storage 15](#__RefHeading___Toc18732)

[3.1.3 Accountability 15](#__RefHeading___Toc32220)

[3.2. Study plan and timing of procedures 15](#__RefHeading___Toc25135)

[3.2.1 Enrolment period (day -14 to day 0): 15](#__RefHeading___Toc29887)

[3.2.2 Randomization and Treatment period (day 0 to 12 months [with maximum of 36 months]): 16](#__RefHeading___Toc27233)

[3.3 Concomitant medications and other treatments 16](#__RefHeading___Toc32183)

[3.3.1 Heart failure medications 16](#__RefHeading___Toc12915)

[3.4 Adverse drug reaction 17](#__RefHeading___Toc9660)

[3.5 Evaluation on compliance 17](#__RefHeading___Toc15961)

[4. Outcome Measures for Analyses 17](#__RefHeading___Toc26695)

[4.1 Clinical observation endpoints 17](#__RefHeading___Toc28063)

[4.1.1 Primary outcome measure 17](#__RefHeading___Toc30945)

[4.1.2 Secondary outcome measures 17](#__RefHeading___Toc22312)

[4.2. Safety outcome measure: 17](#__RefHeading___Toc1630)

[5. Course of Study 18](#__RefHeading___Toc31758)

[6. Efficacy Assessments 19](#__RefHeading___Toc11445)

[6.1. Endpoint reporting overview 19](#__RefHeading___Toc21131)

[6.2 Potential endpoint events 19](#__RefHeading___Toc13012)

[7. Safety Assessment 20](#__RefHeading___Toc28938)

[7.1. Definition of Adverse Event： 20](#__RefHeading___Toc23524)

[7.1.2. Criteria on severity of adverse events: 21](#__RefHeading___Toc8911)

[7.1.3. Adverse events of interest 21](#__RefHeading___Toc4055)

[7.2. Definition serious adverse events 21](#__RefHeading___Toc15064)

[7.2.1 Definition on specificity of serious adverse events 22](#__RefHeading___Toc32053)

[7.3. Recording of adverse events and follow-up 22](#__RefHeading___Toc24302)

[7.4. Adverse events based on examinations and tests 22](#__RefHeading___Toc9859)

[8. Blinding and Unblinding 23](#__RefHeading___Toc5110)

[8.1.1 Random grouping of subjects 23](#__RefHeading___Toc8124)

[8.2. Methods for unblinding 23](#__RefHeading___Toc24060)

[8.2.1. Unblinding provisions 24](#__RefHeading___Toc9394)

[8.3. Screening number 24](#__RefHeading___Toc6560)

[9. Statistical Analysis 24](#__RefHeading___Toc14161)

[9.1. Definitions of analysis sets 24](#__RefHeading___Toc8641)

[9.2. Statistics methods 25](#__RefHeading___Toc11542)

[9.2.1. Enrollment and completion 25](#__RefHeading___Toc11160)

[9.2.2 Compliance analysis: 25](#__RefHeading___Toc3393)

[9.2.3 . Efficacy analysis: 25](#__RefHeading___Toc21672)

[9.2.4. Safety analysis: 26](#__RefHeading___Toc28037)

[9.3. Interim analysis 26](#__RefHeading___Toc31015)

[10. Data Management 26](#__RefHeading___Toc10563)

[10.1. Database Design 27](#__RefHeading___Toc24280)

[10.2. Data entry 27](#__RefHeading___Toc28563)

[10.3. Data questioning management 27](#__RefHeading___Toc24415)

[10.4. Medical coding 27](#__RefHeading___Toc18093)

[10.5. Data audit 27](#__RefHeading___Toc22176)

[10.6. Database locking 27](#__RefHeading___Toc22374)

[11. Quality Control 28](#__RefHeading___Toc31908)

[12. Ethical conduct of the study 28](#__RefHeading___Toc10727)

[13. Study timetable and end of study 29](#__RefHeading___Toc20504)

[14. Data Archiving 29](#__RefHeading___Toc1222)

[15. Clinical Summary 29](#__RefHeading___Toc7531)

[Reference: 29](#__RefHeading___Toc27168)

[1 Appendix 1: New York Heart Association (NYHA) Functional Classification 31](#__RefHeading___Toc20485)

[2 Appendix 2: NT-proBNP test, blood collection, blood sample preservation and transportation process 31](#__RefHeading___Toc19060)

[3 Appendix 3: Endpoint event report process 34](#__RefHeading___Toc26176)

# 1. Introduction

# 1.1 Study Topic

Qiliqiangxin in Heart FailUre：AssESsment of Reduction in MorTality (QUEST study)

# 1.2 Study Purpose

Evidence-based medical research methods are used to explore the clinical efficacy and safety of long-term use of Qiliqiangxin capsule with cardiovascular mortality and incidence of re-hospitalization for recurrence of heart failure as main study endpoints, in order to determine effective characteristics and suitable population, and provide high quality clinical evidence for optimizing clinical drug regimen.

# 1.3 Study Background

Cardiovascular disease is one of the major causes of death globally, seriously threatening human life and health [1, 2]. Chronic heart failure (CHF) is a series of clinical syndromes caused by pump failure, reduced ejection fraction, circulatory congestion and/or a series of neurohormonal changes on the basis of structural and/or functional abnormality [3]. As a serious stage of various heart diseases, epidemiological studies showed that the number of global heart failure patients has reached 22.5 million, and the 5-year survival rate is similar to malignant tumors.

With the changes of epidemiology and the development of social economy, the epidemiological characteristics of heart failure in developing countries are becoming similar with those in developed countries. For instance, coronary heart disease is becoming the main cause of heart failure in China [4, 5]. In recent years, the European Society of Cardiology (ESC) make a statistical study in 51 countries and has found that there are at least 15 million heart failure patients in about 1 billion people. In 2007, the American Heart Association (AHA) reported that the number of heart failure patients has exceeded 5 million in the United States, and is still increasing at a rate of 550,000/year [6]. The incidence is Japan, which has similar geographical location and ethnic group characteristics as China, is similar to that in Europe and the United States. In 2003, GU Dongfeng *et al* randomly surveyed 15,518 adults (aged 35-74 years old) in respective five provinces and cities in the south and north of China, and found that the prevalence of heart failure in China was 0.9%, including 0.7% for men and 1.0% for women [7]. The incidence and prevalence rate are also gradually increasing worldwide; the number of heart failure patients is increasing at a rate of 2 million per year.

Due to the economic improvement, diet change, and also the extending of human life, the incidence of heart failure increases with age [8]. Also, with the development of modern medicine, the mortality rate of patients with heart failure has gradually decreased, but remains in a relatively high level. The 1-year mortality rate of patients with heart failure over 70 years old is significantly higher than that of patients under 70 years old (22%: 13.7%) in the United States [9]. The 1-year and 3-year mortality rates of heart failure patients are 11.3% and 29.2% in Japan, respectively [10]. In Europe, the 4-year survival rate is only 50%, and 40% of heart failure inpatients is admitted to hospital for treatment or dies within 1 year [11, 12]. Chronic heart failure is still a major problem that seriously threatens human life and quality of life which in need to be resolved.

In the past 20 years, the concept of drug treatment for heart failure has changed greatly, from the perspective of improving hemodynamics to the point of biological adjustment. The focus of modern treatment models is to improve the neuroendocrine disorders of the renin- angiotensin-aldosterone system and the sympathetic nervous system. Therefore, the goal of treatment is not only to improve symptoms and quality of life, but also to inhibit and delay the development of myocardial remodeling, terminate the vicious circle, thereby to reduce the mortality and hospitalization rate of heart failure.

Myocardial remodeling is the basic mechanism of heart failure, including pathological cardiomyocyte hypertrophy with re-expression of embryonic genes, cardiomyocyte apoptosis and necrosis, and excessive deposition or degradation of myocardial extracellular matrix. Slowing or preventing myocardial remodeling is of great value in preventing and controlling the occurrence and development of heart failure and improvement of cardiac function. Activation of two neuroendocrine systems, including sympathetic nervous system (SNS) and renin-angiotensin-aldosterone system (RAAS), and myocardial remodeling promote each other and aggravate the development of heart failure. ACEI, beta blockers, ARB and aldosterone receptor antagonists are shown to be beneficial, indicating the effectiveness of inhibiting these two systems. However, the mechanisms of heart failure are not limited to the SNS and RAAS, further research are needed to explore the effective anti-heart failure pathways, and infer new targets for treating heart failure.

Numbers of clinical studies in the late 1980s also confirmed that activation of neuroendocrine system leads to myocardial remodeling, which is a key factor in the occurrence and development of heart failure. In 1987, CONSENSUS, a clinical trial of angiotensin-converting enzyme inhibitor (ACEI) for heart failure, successfully reduced the total mortality of heart failure patients by 27%. Later, clinical trials such as SOLVED and V-HeFY further confirmed that ACEI can effectively improve prognosis of heart failure.

In the mid-to-late 1990s, CIBISII, MERIT-HF and COPERNICUS studies confirmed that beta blockers reduced mortality in heart failure patients by 34% to 35%. In addition, the RALES trial (1999) and EMPHASIS-HF (2011) studies had shown that aldosterone receptor antagonists can reduce mortality in heart failure patients by 24% to 30%.

New drugs that have benefited patients with heart failure since 2010 are mainly ARNI and cardiac sinus node inhibitors. In the PARADIGM-HF trial, 8442 heart failure patients with reduced ejection fraction (HFrEF) were randomized to receive ARNI and enalapril. The incidence of primary endpoints (cardiovascular death and hospitalization for heart failure) was 21.8% in ARNI group, significantly lower than the enalapril group (26.5%) [13]. The SHIFT study showed that the relative risk of hospitalization for cardiovascular death and worsening heart failure was reduced by 18% compared with the standard treatment group, and left ventricular function and quality of life were significantly improved [14] . At the same time, clinical research has also found that diuretics can effectively relieve dyspnea in patients with heart failure. Rational use of diuretics is the key and foundation for other successful treatment of heart failure by eliminates fluid retention, improves heart function and exercise tolerance.

During this period, non-pharmacological treatment of heart failure has also made important progress. Choosing the suitable patient based on drug treatment can further improve the heart function and quality of life and reduce mortality.

Despite some progress in the field of heart failure treatment in these years, the current prevalence and mortality of heart failure remain high. There is still a need to develop new treatments and develop new drugs to achieve breakthroughs in heart failure treatment.

Traditional Chinese medicine (TCM) has accumulated rich experience in the long-term medical practice of preventing and treating heart failure, and its understanding of heart failure is also deepening. There have been some researches and reports on the treatment of heart failure with TCM. The researches on the treatment of heart failure by Chinese medicine QiliQiangxin Capsules was initially successful. The research results were published in an international journal of cardiovascular disease JACC in 2013[15] and received the attention and praise from worldwide scholars. All enrollees were treated with standard-optimized treatment, and the ratio of serum amino-terminal B-type natriuretic peptide precursor (NT-proBNP) was significantly lower in the Qiliqiangxin capsule group compared with the placebo group, higher ratio of patients with a decrease of >30% and lower incidence of adverse events were observed, indicating that Qiliqiangxin Capsules is effective and safe in the treatment of chronic heart failure. Qiliqiangxin Capsules became the first proprietary Chinese medicine to be included in the Guidelines for the Diagnosis and Treatment of Heart Failure in China 2014 and 2018.

Qiliqiangxin Capsules is the first to explore the pathogenesis and treatment of chronic heart failure with the theory of collateral disease, which proposes insufficiency of the heart-qi as the basis of TCM. Meanwhile, obstruction of collaterals is the central link. The fluid does not flow and exudes from veins which the blood stasis and water stasis blocks the veins, inducing edema [16]. The theory is similar with a new concept proposed by modern medicine in recent years that the hemodynamic changes caused by early neuroendocrine activation can lead to ventricular remodeling, which is the basic mechanism for the development of heart failure.

Due to the post-marketing unique advantages of Qiliqiangxin Capsules in the clinical application, it has become a hot spot for many experts and scholars in China. Previous studies have shown that the drug has cardiotonic and diuretic effect, can improve the heart function of CHF rats, and increase water discharge by reducing the expression of AQP2 in the kidney [17]. It also can reduce the expression of AngII and periostin proteins in rats with heart failure after myocardial infarction and is dose-dependent [18]. The mechanism of the effect of Qiliqiangxin Capsules on improving cardiac function is related to its inhibition of myocardial remodeling [19]. Reducing the pro-inflammatory factors of cardiomyocytes and increasing the immunomodulatory effects of anti-inflammatory factors may be one of the immunopharmacological mechanisms of Chinese medicine for improving heart function in AMI rats [20]. In the clinical study of taking Qiliqiangxin Capsules for the treatment of patients with chronic congestive heart failure, it is indicated that Qiliqiangxin Capsules can improve cardiac function grading, heart failure (Lee’s) score, systolic and diastolic function, ejection fraction (EF), TCM syndrome differentiation (中医证候), quality of life and other curative indicators and safety indicators, elevate blood nitric oxide (NO) and calcitonin gene-related peptide (CGRP) levels, and decrease endothelin (ET) levels; thereby significantly improving endothelial function in patients with heart failure.

In summary, Qiliqiangxin Capsules could resolve the current problems and eliminates the essential causes that treat cardiac insufficiency from multiple pathways, multiple links and multiple targets. It reflects the advantages of compound Chinese medicine in treating heart failure from the overall treatment, and significantly reduces NT- ProBNP levels, indicating improvement of the patient's long-term prognosis. This study is a large randomized, double-blind, multi-center clinical study carried out on the basis of achieving major study results with NT-proBNP as a surrogate endpoint, adopts the incidence of composite endpoint events consisting of cardiovascular death and re-hospitalization for heart failure as the study endpoints, in order to collect the evidence-based medical evidences for long-term prognosis in patients with chronic heart failure.

# 2. Study design

# 2.1. Rationale for study design

This study is a randomized, double-blind, placebo-controlled, parallel-group, multicenter clinical study.

The study will be event-driven, and all randomized patients will remain in the study (whether taking the study drug or not) until the number of primary endpoint events reaches the predicted value (620 cases), or the study terminates early when it meets the pre-defined efficacy or safety criteria of early termination.

Two mid-term efficacy analyses planned to be conducted after 1/2 and 2/3 primary endpoint events to assess whether an invalid or valid conclusion was reached so as to prematurely end the study.

The entire study will last approximately 36 months, and the recruitment period will be expected to be 24 months. The follow-up period after the last case of patient is included in the study is 12 months. The average follow-up time is predicted to be about 24 months.

Patients who show stable clinical symptoms, had received at least 2 weeks of standardized treatment and treatment of other concomitant diseases before enrollment are screened at the hospital. According to the local HF treatment guidelines, the drug type and dosage are fixed, unless it is contraindicated or intolerant. The patient who have not receive anti-HF drug intravenously for at least two weeks prior to enrollment, nor take oral administration of TCM or Chinese patent medicine having similar composition with Qiliqiangxin Capsule can directly enter the random grouping stage.

If patients fail to meet the above requirements, term standardized treatment to meet the above criteria before entering the random grouping stage are needed.

# 2.2. Control group and Sample size

According to the PARADIGM-HF study, cardiovascular death or hospitalization rate for heart failure in the median follow-up of 27 months was 21.8% in the LCZ696 group and 26.5% in the Enalapril group. Therefore, we estimate that the incidence of cardiovascular death and hospitalization for heart failure is 25% in all patients of basic treatment +placebo group within 36 months of follow-up and 20% in basic treatment + Qiliqiangxin Capsule group.

The random distribution ratio is 1:1 between study group and control group. Considering the consumption of type I error in the interim analysis, α is adjusted to unilateral 0.02314. The sample size is the number of cases with composite endpoint events. It is expected that 620 composite endpoint events need be observed to provide 80% power of test (β= 0.2), and 20% risk can be reduced in study group by log-rank test.

The entire study will last approximately 36 months to follow up, and the recruitment period are expected to be 24 months. It is expected that 3,080 patients in over 100 centers (1540 patients per group, 1:1 into study group and control group) will be enrolled and be followed up at least for 12 months. Estimating the incidence of composite endpoint events is 25%, in total 620 end-point events are expected to be obtained in the analysis.

# 2.3. Rationale for study population

The enrolled patients should satisfy the following inclusion criteria, and not meet any exclusion criterion. In addition to following criteria, the patient should also be excluded if there is any contraindicated medical condition or use of incompatibility drug during basic treatment period.

# 2.3.1 . Inclusion criteria

1. Provision of signed informed consent prior to any study specific procedures;
2. Male or female, aged ≥18 years at the time of consent;
3. Established documented diagnosis of heart failure for at least three months ago according to “Chinese Heart Failure Diagnosis and Treatment Guideline” issued by the Chinese Medical Association Cardiovascular Branch.
4. Left ventricular ejection fraction (LVEF) ≤40% (echocardiogram, radionuclide, ventriculogram, contrast angiography or cardiac MRI );
5. NYHA cardiac functional grading II to III, with stable clinical symptoms; including those diagnosed as grade IV within 2 weeks before enrollment;
6. Serum NT-proBNP ≥ 450pg/ml;
7. Those who have received standardized baseline treatment regimens without doses adjusted and given intravenously for at least two weeks prior to enrollment;
8. standardized drug treatment includes: angiotensin-converting enzyme inhibitor (ACEI) or angiotensin receptor blocker (ARB) or angiotensin receptor neprilysin inhibitor (ARNI), beta blocker, and aldosterone receptor antagonist (the optimal therapeutic dose should be achieved, except for contraindications or intolerance);

# 2.3.2 . Exclusion criteria

1. Patients should not enter the study if any of the following exclusion criteria are fulfilled
2. Heart failure caused by valvular disease, congenital heart disease, pericardial disease, arrhythmia or non-cardiaogenic disease, or caused by vital organ failure (such as renal, hepatic failure, etc.); and right heart failure caused by pulmonary or other definite causes; and acute heart failure;
3. Coronary revascularization (percutaneous coronary intervention [PCI] or coronary artery bypass grafting [CABG]) or cardiac synchronization therapy planned to undergo after randomization, or had received cardiac resynchronization therapy prior to enrolment;
4. Any condition outside the CV diseases such as but not limited to malignant tumor, severe mental illness, hematopoietic diseases, neuroendocrine system disease, liver transaminase and alkaline phosphatase ≥ 3 x upper limit of normal (ULN), abnormal renal function serum creatinine > 2 mg/dl (176.82 umol/L), potassium >5.5mmol/L;
5. Patient with left ventricular outflow tract obstruction, myocarditis, aortic aneurysm, aortic dissection, or obvious hemodynamic changes caused by unrepaired valve;
6. Cardiogenic shock, uncontrollable malignant arrhythmia, sinus or atrioventricular block at second degree type II or above without pacemaker treatment, progressive unstable angina pectoris or acute myocardial infarction;
7. uncontrolled hypertension systolic blood pressure (SBP) ≥ 180mmHg and/or diastolic blood pressure (DBP) ≥ 110mmHg; or SBP < 90mmHg and/or DBP <60mmHg;
8. Participation in another clinical study with an IP during the last month prior to enrolment;
9. Women of child-bearing potential (i.e., those who are not chemically or surgically sterilized or who are not post-menopausal) who are not willing to use a medically accepted method of contraception that is considered reliable in the judgment of the investigator, from the time of signing the informed consent throughout the study and 4 weeks thereafter, OR women who have a positive pregnancy test at enrolment or randomisation OR women who are breast-feeding;
10. Allergic constitution; known to be allergic to research drug;
11. Inability of the patient, in the opinion of the investigator, to understand and/or comply with study medications, procedures or any conditions may render the patient unable to complete the study.

# 2.4. Patient enrolment and Follow-up visit

Patients who fail to meet the eligibility criteria should not, under any circumstances, be enrolled or receive study medication. Patient should receive at least 2 weeks of standardized treatment. Patient should not receive other traditional Chinese medicine or Chinese patent medicine (with similar function and composition with Qiliqiangxin Capsules).

Patients meet the inclusion criteria can enter the randomization. During this period, the drug and dosage are fixed for each patient. If it is necessary to make adjustment, it should be recorded in the case report form (CRF).

Patients should visit the hospital for efficacy and safety assessment during the 1st, 3rd, 6th, 9th, and 12th months after the random grouping until the study finish.

# 2.5. Discontinuation of investigational product (IP)

At any time after randomization, patients are free to discontinue for any reason. Discontinuation from IP is not the same as complete withdrawal from the study. Study drug treatment can be discontinued when:

1. The patient can stop treatment at any time;

2. The patient has allergic reactions that are clearly associated with the IP;

3. The patient has occurrence of symptoms, signs and/or abnormal examination results that are related to the IP, or the condition determined by the investigator to terminate the study;

4. Pregnancy during the study;

During the trial, the patient should take the standard dose of the IP as long as possible. The patients should resume taking the IP as soon as possible after the relevant causes are excluded and follow up as planned. Generally AEs, SAEs and potential endpoint events should not lead to IP discontinuation, unless there is a clear clinical rationale to do so.

Conversely, patients stopped taking the IP should also participate and complete in the study follow up and evaluation items. Patients that intent to discontinue will always be asked about the reason(s) and the presence of any AEs. If the patient is unable to participate in the study visit, follow-up should be continued by phone as planned to determine if any adverse events and endpoints have occurred unless the patient refuses to follow up and withdraw from the study.

# 2.6 Withdrawal

The patient has the right to withdraw from the study at any time for any reason. The researcher should retrieve the remaining IP when the patient withdraws. The reason for the withdrawal should be acquired by follow-up interview or telephone. Follow-up should be continued in order to ascertain whether any endpoints or safety events have occurred. Optimally, patients who discontinue from IP should continue to attend all study visits according to plan until study finish as much as possible. Information should be recorded in the Case Report Form.

# 2.7. Discontinuation of the study

1. The overall study may be stopped due to the following reasons:

- Base on Data Safety Monitoring Committee (DSMC) interim analysis results;
- Researchers find serious safety problems;
- Major mistakes in the study protocol;
- The sponsors decide to suspend study due to management problems or lack of funding;
- The competent administrative department cancels the experiment, and half-stops all studies.

1. The discontinuation of the study can be temporary or permanent. During the suspension, all study records should be kept for inspection.

# 3. Treatment

# 3.1 Investigational products (IP)

**Study drug:** Qiliqiangxin Capsule (芪苈强心胶囊)

-Ingredients: Astragalus, ginseng, monkshood, Danshen, Pepperweed Seed, rhizoma alismatis, radix polygonati officinalis, cassia twig, red flower, cortex periplocae, tangerine peel

-Property: Capsule; the contents are brown to black brown granules; bitter in taste;

-Specification: 0.3g/ granule

-Bach number: GYZZ Z20040141

-Manufacturer: Shijiazhuang Yiling Pharmaceutical Co., Ltd

**Placebo:** Qiliqiangxin Matching Placebo

-With identical color, specification, packaging, , property of contents and other features with Qiliqiangxin Capsule

# 3.1.1 Package and label

The appearance of small package is shown below and will be labeled with “For QUEST study only”. Each package contains 36 capsules sealed in aluminum-plastic plates.


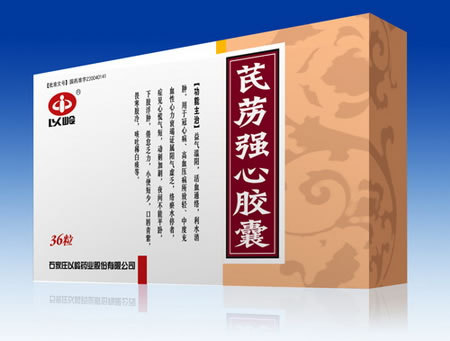


Big package: White paper box, with size of 29.5cm╳12cm╳22cm, each big package includes 33 small packages.

Each is marked with following label:

| **Qiliqiangxin in Heart FailUre: AssESsment of Reduction in MorTality (QUEST)**  **(For Clinical Study Only)** |
| --- |
| **Package No.: XXXXX**  [Lot number] xxxx [Expiry date] xxxx |
| [GYZZ] Z20040141  [Pakcage] 33 small boxes, and 36 granules in each small box (amount for 99-days)  [Directions] Three times a day and four granules each time  [Storage] Sealed in a cool dry place. Please store the product out of children's reach. |
| **Please be sure to follow doctor's orders and visit at the specified date to the hospital for follow-up evaluation. Thank you for your cooperation!** |

# 3.1.2 . Storage

All IP should be kept in a secure place under appropriate storage conditions. Each center must assign a committed staff to preserve and manage the study drug.

# 3.1.3 Accountability

The IP provided for this study will be used only as directed in the study protocol. Patients will be asked to bring all unused study medication and empty packages to the study. During each follow-up, the investigator or delegate will collect and check the amount of returned capsule and fill in Drug Distribution Form, timely and accurately to account for all IP dispensed to and returned from the patient, in order to determine the compliance of subjects at each site visit.

At the end of study, all remaining drugs should be returned. Undistributed IPs should be sealed when returned. Remaining drugs should be retained and uniformly destroyed after study.

During each follow-up, the subjects should return all remaining drugs, and researchers must make an inventory of remaining drugs and keep record

# 3.2. Study plan and timing of procedures

# 3.2.1 . Enrolment period (day -14 to day 0):

The investigators review the inclusion and exclusion criteria. Patients who do not meet these criteria must not be randomized into the study. During enrolment period the following assessments and procedures will be completed.

-Demography (date of birth, sex, race, ethnic group) and relevant medical and surgical history, including smoking history, will be recorded.

-General physical examination (vital signs, NYHA classification, appearance, cardiovascular systems [including edema], etc.)

-Laboratory samples will be collected and sent to the central laboratory

-ECG and echocardiogram will be recorded

# 3.2.2 Randomization and Treatment period (day 0 to 12 months [with maximum of 36 months]):

The dispensing date of the IPs is regarded as day 0. Patients will be randomized into the study or control group in a 1:1 ratio w

ith the basis of current standardized treatments for chronic heart failure. The study drug is recommended to be taken about 30 minutes after meals, three times a day as follow.

-Study group: Standardized treatment of chronic heart failure + Qiliqiangxin Capsules (4 capsules/time, 3 times/day);

-Control group: Standardized treatment of chronic heart failure + Placebo Capsules (4 capsules/time, 3 times/day);

This study does not allow dose adjustments. If the patient miss to take the IP in a day, the accumulated dose for the next day should not exceed the daily dose. If the patient has an intolerable adverse event, which is relevant to study drug according to the judgment of researcher, the patient should terminate the following treatment with the study drug.

Do not take drugs at home on the morning of visit day. Investigators will review laboratory results received from the past visit(s). If the patient has experienced any potential endpoints, SAEs, DAEs and/or AEs of interest since the last visit, these should be recorded in the CRF.

# 3.3 Concomitant medications and other treatments

All patients should be treated according to regional standard of care for HF and other comorbidity(s). Also, cardiac and heart failure related procedures will be captured during the study. Background medication will not be provided by the Sponsor.

1. Detailed recording of medications related to HF, HTN as well as other relevant cardiovascular medications (e.g., statins, antihypertensive and antithrombotic agents) will be made throughout the study;
2. Traditional Chinese medicines have similar contents to the test drug should not be used during the entire treatment period after entering the randomization period;
3. Patients should receive dietary guidance for heart health, such as low-salt diet, moderate drinking, etc. Patients should also receive counseling for appropriate lifestyle improvements such as weight monitoring, physical exercise, smoking cessation, and alcohol withdrawal.
4. No drug has been found to be prohibited with use of Qiliqiangxin Capsules.

**3.3.1 Heart failure medications**

According to local HF treatment guidelines, or the “Chinese Heart Failure Diagnosis and Treatment Guide 2018” issued by the Chinese Medical Association Cardiovascular Branch, the specifications are as follows:

1. Patients should remain on stable doses of medications which will allow assessment of incremental QLQX effect. No intravenous injection of diuretic, cardiac stimulant or vasodilator substance at least two weeks before enrollment;
2. Patient should receive standardized drug treatment of chronic heart failure at least two weeks before randomization grouping stage, and all drugs should be adjusted to fixed dosage. Standardized drug treatment includes: angiotensin-converting enzyme inhibitor (ACEI) or angiotensin receptor blocker (ARB) or angiotensin receptor neprilysin inhibitor (ARNI), beta blocker, and aldosterone receptor antagonist (the optimal therapeutic dose should be achieved unless contraindicated or not tolerated);

3) After entering the treatment period, Dose reduction or discontinuation of proven effective therapies should be avoided unless all other measures fail to improve the patient’s situation.. If there is any need for adjustment, reason and regimen change should be recorded in the CRF.

# 3.4 Adverse drug reaction

Adverse reactions to test drugs are not known. The National Adverse Reaction Monitoring Database showed the possible reactions might included gastric discomfort such as nausea, bloating, and hiccups, etc.

# 3.5 Evaluation on compliance

In order to determine the compliance of subjects, the administration (drug distribution and recovery) of all investigational products should be recorded in the appropriate sections of the eCRF.. The actual dosage should be within 80%-120% of predefined dosage.

# 4. Outcome Measures for Analyses

# 4.1 Clinical observation endpoints

# 4.1.1 . Primary outcome measure

- - - The composite endpoint events consisting of cardiovascular death
    - Re-hospitalization due to the worsening of heart failure;

# 4.1.2 . Secondary outcome measures

- All-cause mortality
- Secondary endpoint events (given up treatment due to worsening heart failure, successful resuscitation after cardiac arrest, malignant arrhythmia, non-fatal stroke)
- Cardiovascular death and re-hospitalization due to worsening heart failure in patients with ischemic heart disease
- Level of Serum NT-proBNP

*Note: All endpoint events should be determined and reviewed by Clinical Event Adjudication Committee.*

# 4.2. Safety outcome measure:

- Adverse events (Serious Adverse Events [SAEs], Discontinuation of IP due to Adverse Events, etc.)
- AEs of interest (volume depletion, renal events, etc.)
- Clinical laboratory indexes: blood routine test (hemoglobin, red blood cells, white blood cells, platelets), routine urine test (urinary protein, urinary white blood cells, urine red blood cells), serum biochemistry (urea nitrogen, creatinine, blood uric acid, total protein, albumin, alanine aminotransferase, aspartate aminotransferase, alkaline phosphatase, total bilirubin, fasting blood glucose, potassium, sodium, chlorine, total cholesterol, triglycerides, low density lipoprotein, high density lipoprotein).
- 12-lead ECG
- Physical examination

# 5. Course of Study

All participants, including those discontinue study drug prior to completion of study, should continuously take all planned visits listed in the table until the end of study. If a visit is postponed or taken in advance, it should not affect the next visit. The next visit should be carried out in accordance with the original planned time.


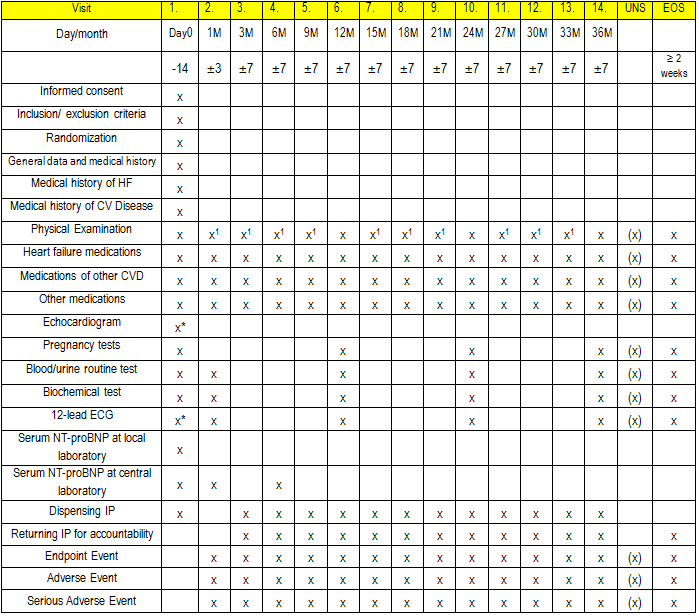
×¹ Simplified physical examination

- ×* Cardiac ultrasound and 12-lead ECG within the first 6 months of enrollment;
- UNS (unplanned visit): (x) The marked item is optimal and performed according to the judgment of researchers.
- EOS (final visit): Make arrangement according to the study end time (if there is a visit within one month before the end of study, it is regarded as a final visit, but needs to be supplemented with the items required completely)
- Pregnancy test is only applicable to women of childbearing age (if the urine pregnancy test is positive, it must be confirmed by serum pregnancy test).

# 6. Efficacy Assessments

# 6.1. Endpoint reporting overview

When potential endpoint events have been identified, the researchers should collect all relevant support documents within 7 days and report to CEA committee. Investigators will record the incident in endpoint report form and submit supporting data in a timely manner (admission and discharge records, medical records, death records, ECG, etc.). The potential endpoints event (All deaths, All HF events [hospitalizations for HF or urgent HF visits], cardiac ischemic events [MI and unstable angina], cerebrovascular events [stroke and TIA], etc.) will be reviewed for central CEA process.

CEA committee consists of a chairman and 5-6 members. Each case will be independently reviewed by two members of the committee. The conclusions will be submitted to the chairman of the committee.

# 6.2 Potential endpoint events

For each potential endpoint event, the investigator or delegate will record information in the CRF

- **Hospitalization for heart failure:**

1. The patient was hospitalized for HF diagnosed preliminarily;

2. The patients who were in hospital extended at least 24 hours (or if the hospitalization time and discharge time were not available, it should indicate change of calendar date);

3. Record on the patient report that there are new symptoms or worsening symptoms due to HF, including at least one of the following:

a. Difficulty breathing (difficulty breathing on exertion, difficulty breathing on resting, orthopnea, paroxysmal breath with difficulty at night)

b. Reduced exercise tolerance

c. Fatigue

4. The patient had objective evidence of an acute exacerbation of HF, including at least two health examination results or a health examination result and at least one laboratory standard, including:

a. Determine the health examination results caused by HF, including new or deteriorated:

1) Peripheral edema; 2) Abdominal distension or increase of ascetic fluid (in the absence of primary liver disease); 3) Lung rales and/or crackles; 4) Increased jugular venous pressure and/or hepatojugular reflex (+); 5) S3 galloping; 6) Clinically significant or rapid weight gain, having relation with fluid retention;

b. Laboratory evidence of new or worsening HF obtained within 24 hours, including:

1) Increased concentration of B-type natriuretic peptide (BNP) / N-terminal B-type natriuretic peptide precursor (NT-proBNP) consistent with acute decompensated HF (eg.: BNP > 500 pg/ml or NT-proBNP > 2000 pg/ml); In patients with long-term elevation of natriuretic peptides, special attention should be paid to a significant increase beyond baseline. 2) Imaging evidence of pulmonary congestion; 3) Non-invasive diagnostic evidence of clinically significant increase in left or right ventricular filling pressure or decreased cardiac output; echocardiographic criteria includes: E/e ́>15 or D leading pulmonary venous inflow pattern, congestive inferior vena cava with minimal inspiratory collapse, or reduction of small stroke distance (time velocity integral; TVI) at left ventricular outflow (LVOT). 4) Invasive diagnostic evidence: right heart catheterization showed pulmonary capillary wedge pressure (pulmonary occlusion pressure) ≧ 18mmHg, central venous pressure ≧ 12mmHg, or cardiac output index;

*Note: If applicable, all results in the diagnostic check need to be reported; even if the above criteria are not met, results might provide important information for the determination of the above events.*

5. The patients receive an initial or intensive treatment for HF, including at least one of the following:

a. Enhance the treatment of oral diuretics;

b. Intravenous diuretics or vasoactive drugs (such as positive inotropic drugs, vasopressors or vasodilators);

c. Mechanical or surgical intervention, including:

1) Mechanical circulation support (e.g.: Intra-aortic balloon pump, ventricular assist device, extracorporeal membrane oxygenation, total artificial heart);

2) Mechanically assisted removal of body fluids (e.g.: ultrafiltration, hemofiltration, and dialysis);

- **All-cause mortality:** For the purpose of the efficacy analysis, death will be subclassified and recorded as CV or Non-CV death in the CRF
- **Cardiovascular death:** including death caused by acute myocardial infarction (AMI), sudden cardiac death, acute decompensated heart failure, stroke, cardiovascular (CV) surgery, CV bleeding, and other CV inducing death;
- **Given up treatment due to the worsening of HF:** Worsening of heart failure symptoms and signs, requiring intravenous drug or mechanical support treatment, and patients or family members voluntarily give up treatment or left hospital without cure; if the result of follow-up is death, it is included in heart failure death.
- **Successful resuscitation after cardiac arrest**
- **Malignant arrhythmia：**There is no uniform standard for the definition of malignant arrhythmia. It generally refers to arrhythmia that can cause severe hemodynamic disorder in a short period of time, causing syncope or even sudden death. According to this standard, malignant arrhythmia mainly has the following categories: (1) severe bradyarrhythmia, such as severe sick sinus syndrome, high or third degree atrioventricular block; (2) tachyarrhythmia, such as persistent ventricular tachycardia, ventricular flutter, ventricular fibrillation, atrial flutter/atrial fibrillation with rapid ventricular rates, atrioventricular reentry tachycardia, pre-excitation syndrome with atrial fibrillation, sinus tachycardia, etc.
- **Non-fatal stroke**

# 7. Safety Assessment

# 7.1. Definition of Adverse Event：

- Adverse events (AE): AE refers to any adverse medical events occurring in this clinical experiment from the moment that the patient signs the informed consent and is chosen to participate in this study to the last follow-up, whether or not the events are caused by the use of the medicine described.

# 7.1.2. Criteria on severity of adverse events:

All adverse events during this clinical study should be recorded in Adverse Event Page of CRF. The severity of adverse events is classified with a uniform standard:

Mild With perceptible discomfort but not affect daily activity

Moderate With marked discomfort that affects daily activity

Severe Unable to work or perform daily physical activity without discomfort

Severity is a measure of intensity. An AE of severe intensity need not necessarily be considered as serious adverse events (SAE). For example, as headache may be severe on the intensity, but cannot be regarded as SAE, unless meeting SAE criteria.

# 7.1.3. Adverse events of interest

The causal judgment between all adverse events and study drugs should be determined and classified by five grades: affirmative relevance, probable relevance, possible relevance, suspected relevance and impossible relevance. The former three are regarded as adverse drug reaction (ADR).

The following factors should be considered:

1. Whether or not there is logical connection between the initiation of medicine treatment and the appearing time of suspected ADR (usage of drug);
2. Whether or not the suspected ADR is related to other known medicine (accord with literature and/or drug instruction);
3. Whether or not the suspected ADR could be explained by the patient’s pathological situation, combined medication, combined or former therapy, etc. (other explanation);
4. Whether or not the suspected ADR is alleviated or disappeared after the drug withdrawal or dose reduction (withdrawal remission);
5. Whether or not the same reactions reoccur after the patient taking the suspected medicine again (reoccur after reuse);

Researchers should evaluate possible associations between adverse events and study drug and combined drug; with reference to the following table:

| Consideration | Usage of drug | Consistent with literature | Other explanation | Withdrawal remission | Reoccur after reuse |
| --- | --- | --- | --- | --- | --- |
| Affirmative relevance | ＋ | ＋ | － | ＋ | ＋ |
| Probable relevance | ＋ | ＋ | － | ＋ | ？ |
| Possible relevance | ＋ | ＋ | ± | ± | ？ |
| Suspected relevance | ＋ | － | ± | ± | ？ |
| Impossible relevance | － | － | ＋ | － | － |

# 7.2. Definition serious adverse events

 Significant adverse events (SAE) refer to any adverse event requiring pertinence medical measures (as withdrawal, dose reduction and symptomatic treatment) and hematologic abnormity and /or other laboratory abnormity.

An SAE is an AE occurring during any study phase (i.e., run-in, treatment, washout, follow-up), that fulfils one or more of the following criteria:

- Death
- At life-threatening condition (the patient with this event has risk of immediate death at the time of occurring events; but do not include the aggravation of events that can lead death)
- With required hospitalization or extended hospital stay
- Sustained or significant loss of productivity or disability
- Congenital malformation

Other condition that may jeopardize the patient or may require medical intervention to prevent one of the outcomes listed above can also be classified as SAE.

# 7.2.1 . Definition on specificity of serious adverse events

In this study, the following events will not be reported as serious adverse events unless they are judged negative and the researcher believes that it is related to the study drug

- Cardiovascular death
- Re-hospitalization for heart failure
- Given up treatment due to worsening heart failure
- Successful resuscitation after cardiac arrest
- Malignant arrhythmia
- Non-fatal stroke

Other events leading to fatal outcomes should be reported as serious adverse events.

# 7.3. Recording of adverse events and follow-up

If any adverse events occurred, especially those associated with the study drug, should be followed up until the patients return to baseline or tend to stabilize. If the baseline status or stability cannot be restored after follow-up, it should be noted in the CRF. All SAEs have to be reported within 24 hours, whether or not considered causally related to the investigational product, or to the study procedure(s).

# 7.4. Adverse events based on examinations and tests

The results from protocol mandated laboratory tests and vital signs will be summarized and give possible interpretation. The abnormal laboratory results caused by reported adverse events should be recorded in the adverse event form. The abnormal results with clinical significance that meets one or more following conditions should be recorded as independent diagnosis on adverse event page of CRF (excluding abnormal laboratory result caused by reported adverse events):

- With associated clinical signs and symptoms
- Change in course of study drug’s treatment dose
- Change in any of standard evidence based medications and (or) other treatment measures need to be changed

# 8. Blinding and Unblinding

**8.1 Methods for ensuring blinding**

The blinding of treatment is ensured by using a double-blind technique. The Qiliqiangxin capsules and the respective placebo capsules will be identical in size, colour, smell, and taste. The bottles with IP will be labelled with unique identification numbers.

# 8.1.1 Random grouping of subjects

Statistical experts at Peking University Clinical Research Institute adopts SAS 9.4 statistical software package to generate random numbers using the block randomization method according to the ratio of 1:1 between study group and control group. The study drug (Qiliqiangxin or placebo capsules) was packaged according to this random number by the person unrelated to the study.

A randomization and trail supply management system (RTSM) is used in the study, and statistical professionals will provide a random numbered list to the RTSM. The patient is then assigned a random number by the RTSM.

After completing baseline assessment, random numbers are assigned by RTSM during baseline visits. After that, the drug number is obtained through the RTSM according to the interview plan, and the serial number of drug assigned each time is different, but the drugs are the same. Before randomization of patient, the researcher must first log into the RTSM and provide the according information (e.g. the subject's date of birth, gender).

# 8.2. Methods for unblinding


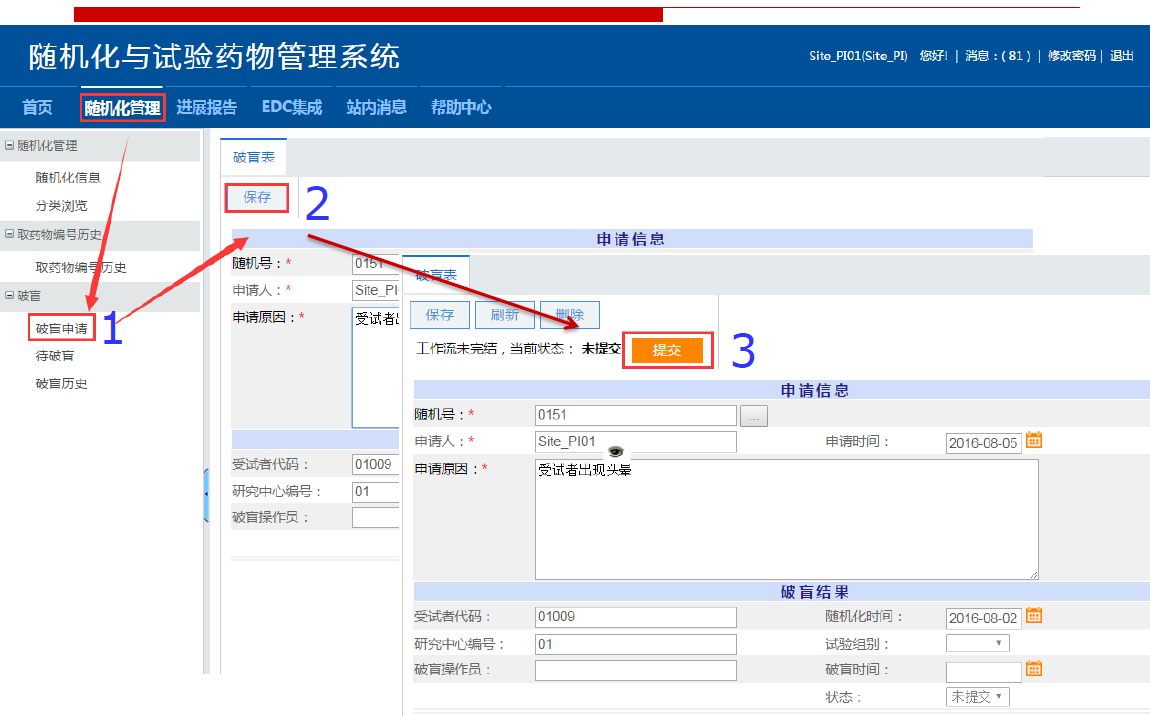
Individual treatment codes, indicating the randomised treatment for each patient, will be available to the investigator(s) or pharmacists from the RTSM in case of adverse events. When the appropriate management of the patient requires knowledge of the treatment randomization, emergency unblinding can be proceeded. Researchers need to login RTSM and fill out the unblinding application, which would be reviewed by the main investigator. If unblinding is deemed necessary, the investigator can perform the unblinding in RTSM and must document all actions taken. Then, unblinding could be proceeded by the unblinding applicant. Clinical data for the above mentioned events will be evaluated and recorded as withdrawal case or AEs/SAEs.

# 8.2.1. Unblinding provisions

All personnel involved with the analysis of the study will remain blinded until database lock and protocol violations have been identified and documented The study adopts two-step unblinding provision. After blind check, the data is locked, main researchers, medical statisticians, data administrators and sponsor representatives will do the first unblinding, and the random number corresponding to the group will be marked as A or B, in order to make statistical analysis on all data. At the end of statistical analysis when summary report is completed, a secondary unblinding would be taken to reveal the group of A and B.

# 8.3. Screening number

The screening number was made according to the sequence of patients taking treatment at each hospital, and represented with center ID + three integers, such as the screening number is 01001, 01002 ….. for center 01.

# 9. Statistical Analysis

After determining study program, statistical professionals should take responsibility of formulating statistical analysis plan after consulting with main researchers. Sample size is estimated by PASS13. SAS®9.4 software (or higher version) is adopted for statistical analysis.

# 9.1. Definitions of analysis sets

- Full Analysis Set (FAS): refers to the data set obtained by removing the subject with the least intentional and reasonable methods from all randomized subjects as close as possible to the intention to treat. It contains all subjects who have been randomized and used study drug once. Exclusions usually included violations of important inclusion criteria, subjects not receiving treatment with the study drug, no observations after randomization. The main efficacy evaluation index was the time when the composite endpoint event occurred, and the survival analysis method was adopted for analysis. When the FAS was selected for statistical analysis, the loss of the primary endpoint event was treated according to the censorship.
- Per Protocol Set (PPS): A subset of the full analysis set, and these subjects are more compliant with the program. Subjects included in the PPS generally have the following characteristics: (1) Take the minimum exposure of study drug set in advance, that is, the compliance of taking the drug is 80%; (2) Main indicators are available in the study; (3) There was no major violation of the study protocol.
- Safety Set (SAS): All subjects who received at least one treatment after randomization and had a safety assessment. Security missing values do not need to be carried over.

# 9.2. Statistics methods

Efficacy analysis is taken on the basis of FAS and PPS. All baseline demographic data analysis will be performed on the basis of FAS and safety evaluation on SAS.

- All data are performed with two-sided test, and P value less than or equal to 0.05 (two-sided test) is considered with statistical difference (unless otherwise specified).
- Descriptive analysis: Classification data is described with number of cases and percentage. Quantitative data is described with mean, standard deviation, maximum value and minimum value, median, the first quartile (Q1) and third quartile (Q3).
- Comparison of general situation should be analyzed with appropriate method based on the type of index. Quantitative data should be analyzed with paired t test or Wilcoxon rank sum test; classification data with chi-square test or precise probability method, and grade data with Wilcoxon rank-sum test or CMH test.

# 9.2.1. Enrollment and completion

The following information would be initially analyzed:

- Summarize the number of enrollment and completion of each center;
- Detailed list for the number of falling off and withdrawal cases, and the total abscission rate;
- Group size in different data sets;
- Case distribution of each center
- Demographic characteristics (age, height, weight, vital signs, etc.), medical history and medication history between groups

# 9.2.2 Compliance analysis:

- Medication compliance analysis: comparison between two groups of the investigational drug intake condition (e.g. timely and correct intakes, prohibited medicine and food of the scheme, etc.)
- Drug combination analysis: analysis for the detailed list of drug combination in each group;

# 9.2.3 . Efficacy analysis:

- PP analysis and FAS analysis were performed simultaneously with efficacy evaluation;
- Main efficacy evaluation index is the time when a composite endpoint event (cardiovascular death and re-hospitalization for deterioration of heart failure) occurs. The lack of primary endpoint events is considered as censored data.

The main research hypotheses are:

H0: λT / λC ≥ 1

H1: λT / λC < 1

λT and λC are the risk of endpoint events in the study group and control group respectively. The Kaplan-Meier method was used to estimate the incidence of clinical endpoints, and a Log rank test was performed for analysis between the two groups. COX proportional hazard model was used and the center was served as a covariate to estimate the hazard ratio and the 95% confidence interval. The composite endpoints, cardiovascular death and rehospitalization for deterioration of heart failure, were analyzed separately.

- - Secondary efficacy indicators:
    - All-cause mortality
    - Composite endpoints (given up treatment due to worsening heart failure, successful cardiac arrest after resuscitation, malignant arrhythmia, non-fatal stroke)
    - Cardiovascular death and re-hospitalization for decompensated heart failure in patients with coronary heart disease
    - Serum NT-proBNP: statistical analysis of measurement data between groups.

# 9.2.4. Safety analysis:

Safety analysis is taken based on SS data set.

Data of adverse events (case number, times and incidence of various adverse events) are compared between the two groups. At the same time, detailed description of specific manifestation, extent of all adverse events and the relation with drugs would be further analyzed.

Crosstab is adopted to describe the change of laboratory index. Number of normal cases before treatment, number of abnormal cases after treatment and ratio of abnormal cases are analyzed in study group and control group. Indexes of vital signs are compared between before and after treatment.

# 9.3. Interim analysis

The study plans to perform two interim efficacy analyses after collecting 1/2 and 2/3 primary endpoints to assess whether a valid conclusion has been reached and then terminate the study early. According to Lan-DeMets α spending function and the O'Brien-Fleming method, the spending type I error was α = 0.0001 (one side) in the first interim analysis period, and α = 0.00605 (one side) in the second interim analysis period.

The specific requirements and operations related to the interim analysis will be specified in the DSMB in advance.

# 10. Data Management

This study used Epidata software to collect research data. Data management ensures the authenticity, integrality and accuracy of clinical data. The data management process needs to comply with the regulatory requirements of Clinical Trial Quality Management Regulations and Clinical Trial Data Management Work Technical Guidelines, in order to ensure traceability of study data. The main processes for data management are listed below.

# 10.1. Database Design

The data administrator adopts the Epidata software to design and release database according to the CRF after testing.

# 10.2. Data entry

Clinical research coordinator (CRC) is responsible for inputting the CRF data into the database. The data entry adopts secondary recording mode. Two CRC respectively input the data. Data administrator compares the two databases to generate the data inconsistency list. CRC modified the databases respectively according to the list and the CRF, and then made comparison again. The above steps are repeated until the two databases being identical.

# 10.3. Data questioning management

The data administrator wrote data verification SAS program according to the data verification plan (DVP) to verify and generated a data questioning list. The data questioning table would be generated after manual verification, and clinical research auditor (CRA) gives the data questioning table to the researcher for answer. After the researcher answering the question, CRA returned the data questioning table to data administrator and revised the database accordingly.

# 10.4. Medical coding

The medical coding of adverse events is done according to MedDRA 21.0 or advance version.

# 10.5. Data audit

After completion of database cleanup, the data administrator should write Data Verification Report for holding a data verification meeting.

The major recording contents of the audit report: number of enrolled cases, the condition of off cases and exclusion cases, the condition of deviation or violation from the program, compliance data, drug combination, adverse events, data related to the evaluation indicators, etc.

At the data audit meeting, the division of statistical population is discussed and determined according to the content of audit report.

# 10.6. Database locking

Complete detailed list of database locking and complete the database locking according to the program of database locking. Problems discovered after data locking can be corrected in the statistical analysis program after confirmation. After data locking, if there is clear evidence that it is necessary to unlock, the researcher and related personnel need to sign the unlocking document. After database locking, the data file is exported by the data administrator and sent to the statistician for statistical analysis.

# 11. Quality Control

1. Main research units and researchers should perform respective duties, strictly abide by clinical research programme, adopt standard operating procedure, verify all the related observed result, find to guarantee the quality control of clinical research, and the implementation of quality assurance system.
2. The subjects in clinical research should be distributed according to random allocation program decided by research design and the block encoding of every subject should be saved by main research units and researcher as blind codes.
3. Researcher should make necessary training, explaining related information, operation standard and responsibility to all the people participating clinical research as well as guarantee to record data into case history and CRF with sincerity, accuracy, integrity, timeliness and legitimacy. CRF must be saved by specially assigned person. Participant should own the qualification certificate of project training.
4. The supervisors should take reference to standard operation procedure, supervise and urge the execution situation of research programme, affirm correctness and integrity of all data records and reports. All CRF should be filled in correctly and keep consistency with original material.
5. The sponsors entrust auditors with systematic examination on relevant clinical activity and documents to evaluate whether the study is conducted in accordance with protocol, standard operating procedure and relevant regulatory requirement or not.
6. Various laboratory inspection data in clinical research should be recorded accurately or pasted the original report copy on CRF.
7. Medical statistician should bring research data into report timely, entirely and inerrably, all steps involved in data management should be recorded to make inspection on data quality and research implementation.
8. Statistical analysis process and the expression of results of clinical research material should adopt standard statistics method. Medical statistician should participate in every phase of clinical research. Statistical report of clinical research should conform to final report of clinical research.
9. All parties should follow with approved trial program, any situation deviating from the program should be recorded. The modification of research program should formulate modification description and be carried out after submitting to Ethics Committee for approval.
10. Each study center should consist of a study principal and several permanent researchers. The study should be conducted in strict accordance with clinical study program. The technical staff in head unit should maintain close contact with the research centers at any time, and visit each study center at early, middle and late stages of the study to inspection and timely resolve any possible problem.

# 12. Ethical conduct of the study

1. The rights, interests and safety of subjects should be considered prior to any science and social interest. The personal interests of subjects should be given sufficient protection and through the entire process.
2. Research program can only be implemented after deliberation agreement and signature of approval opinion by Ethics Committee. Any modification to research program should be approved by Ethics Committee during the period of research; timely report should be submitted to Ethics Committee if serious adverse event was occurred in research.
3. Researcher or the appointed representative should state the detailed research situation to all subjects. Informed consent should be acquired after sufficient and detailed explanation to research situation.

# 13. Study timetable and end of study

| July 2018 | Complete development of study programme, and hold preparatory meeting |
| --- | --- |
| September 2018 | Modify plan and pass ethical audit |
| October 2018 | Study drug and data preparation |
| January 2019 | International registry of the study and start-up of sub-centers |
| March 2019 | Select and enroll the first case |
| October 2020 | Complete randomized grouping of all cases |
| October 2021 | Complete follow-up of all cases in each center |
| December 2021 | Complete data entry and blind review |
| April 2022 | Statistical analysis |
| June 2022 | Complete the study summary report |

# 14. Data Archiving

All study hospitals should keep these original data at least for five years after the termination of clinical study, including confirmation of all participants (effectively verify all records, such as CRF and hospital original record), informed consent, CRF form, and detailed records of drug distribution of all subjects.

# 15. Clinical Summary

After the end of statistical analysis, the main researchers are responsible for composing the clinical summary report and affixing the official seal of main research unit.

## Reference:

1. Lopez AD. Assessing the burden of mortality from cardiovascular diseases [J]. World Health Star Q, 1993, 46(2):91-96.
2. Cardiovascular Branch of Chinese Medical Association. Guidelines for the diagnosis and treatment of chronic heart failure [J]. Chinese Journal of Cardiovascular Diseases, 2007, 35(12):1076-1095.
3. Yuzhu CHEN. Practical Internal Medicine [M]. Beijing: People's Medical Publishing House, 2001.
4. World health organization. Cardiovascular disease: prevention and control [EB/OL]. http://www.who.int/dietphysicalactivity/ publications/facts/cvd/en/. 2010-03-07.
5. Mendez GF, Cowie MR. The epidemiological features of heart failure in developing countries:a review of the literature[J]. Int J Cardiol, 2001, 80(2-3):213-219.
6. Rosamond W, Flegal K, Friday G, et al. Heart disease and stroke statistics - 2007 update - A report from the American Heart Association Statistics Committee and Stroke Statistics Subcommittee[J]. Circulation, 2007, 115(5):E69­E171.
7. Dongfeng GU, Guangyong HUANG, Xigui WU, et al. Epidemiological investigation of heart failure in China and its prevalence rate [J]. Chinese Journal of Cardiovascular Diseases, 2003, 31(1):3-6.
8. Roger VL, Weston SA, Redfield MA, et al. Trends in heart failure incidence and survival in a community-based population[J]. JAMA, 2004, 292(3):344-350.
9. Pulignano G, Del S D, Tavazzi L, et al. Clinical features and outcomes of elderly outpatients with heart failure followed up in hospital cardiology units: data from a large nationwide cardiology database (IN-CHF Registry).[J]. American Heart Journal, 2002, 143(1):45-55.
10. Kawashiro N, Kasanuki H, Ogawa H, et al. Clinical characteristics and outcome of hospitalized patients with congestive heart failure: results of the HIJC-HF registry [J]. Circulation Journal Official Journal of the Japanese Circulation Society, 2008, 72(12):2015.
11. Stewart S, Macintyre K, Hole D J, et al. More 'malignant' than cancer? Five-year survival following a first admission for heart failure.[J]. European Journal of Heart Failure, 2014, 3(3):315-322.
12. Cowie M R, Wood D A, Coats A J, et al. Survival of patients with a new diagnosis of heart failure: a population based study.[J]. Heart, 2000, 83(5):505-10.
13. Mcmurray J J, Packer M, Desai A S, et al. Angiotensin-neprilysin inhibition versus enalapril in heart failure.[J]. New England Journal of Medicine, 2014, 371(11):993-1004.
14. Swedberg K, Komajda M, Böhm M, et al. Ivabradine and outcomes in chronic heart failure (SHIFT): a randomised placebo-controlled study.[J]. Lancet, 2010, 376(9758):2069-2069.
15. Xinli LI, Jian ZHANG, Jun HUANG, et al. A multicenter, randomized, double-blind, parallel-group, placebo-controlled study of the effects of qili qiangxin capsules in patients with chronic heart failure [J]. Journal of the American College of Cardiology, 2013, 62(12):1065-1072.
16. Yiling WU. Meridian theory [M]. China Science and Technology Press, 2010.
17. Dingli XU. Effect of Qiliqiangxin Capsules on cardiac function and renal aquaporin-2 in rats with chronic heart failure. 2009 Xi'an National Heart Failure Academic Conference.
18. Jiazhen LI, Ping YANG. Study on intervention effect of Qiliqiangxin Capsules on periostin protein expression in rats with heart failure after myocardial infarction [J]. Chinese Journal of Laboratory Diagnosis, 2009, 13(2):170-172.
19. Ya LI, You SONG, Xiang CHENG, et al. Effects of Qiliqiangxin Capsules on myocardial remodeling and cardiac function after myocardial infarction in rats [J]. Chinese Journal of Molecular Cardiology, 2007, 7(4):201-204.
20. You SONG, Ya LI, Xiang CHENG, et al. Effect of Qiliqiangxin Capsules on regulation of TNF-α and IL-10 expression in myocardium of rats with acute myocardial infarction [J]. Chinese Journal of Immunology, 2007, 23(9):806-810.

# Appendix 1: New York Heart Association (NYHA) Functional Classification

| **NYHA Class** | **Symptoms** |
| --- | --- |
| I | No limitation of physical activity. Ordinary physical activity does not cause undue fatigue, palpitation, dyspnea (shortness of breath). |
| II | Slight limitation of physical activity. Comfortable at rest. Ordinary physical activity results in fatigue, palpitation, dyspnea (shortness of breath). |
| III | Marked limitation of physical activity. Comfortable at rest. Less than ordinary activity causes fatigue, palpitation, or dyspnea. |
| IV | Unable to carry on any physical activity without discomfort. Symptoms of heart failure at rest. If any physical activity is undertaken, discomfort increases. |

# Appendix 2: NT-proBNP test, blood collection, blood sample preservation and transportation process

**Blood sample preparation test and standard operating procedures of cold chain transport**

**1． Purpose**

Standardize the preservation, transportation and reception conditions and processes of blood samples

**2．Scope of application**

Blood sample preparation, test and transportation in a randomized, double-blind, placebo-controlled multi-center clinical trial to evaluate the efficacy and safety of Qiliqiangxin Capsules in the treatment of chronic heart failure;

**3．Simple process**

Blood sample preparation

Storage Responsible party (researcher)

Handover

Transportation Responsible party (express company)

Receive

Laboratory

**4．Preparation and storage**

The blood sample should be finished preparation within 2 hours after collection according to the standard operating procedures and storage of blood samples. The blood samples should be placed in the freezer tubes respectively. Attach two sheets of the adhesive sticker to the freezer tube according to the number of the drug and the other sheet to the log sheet of reserved sample (Table 1) and fill in the log sheet of reserved sample as required.

**NT-proBNP** **sample preparation process**

Draw 3ml venous blood from each subject

EDTA anticoagulation

Centrifuge to obtain the supernatants

Label the blood samples of each patient

Storage in the refrigerator at -20℃

All samples should be sent for test within 3 months after collection

Note:

1. It should be detected in the condition of no inflammation nor infection (metabolic stability) to reduce individual differences;
2. Patient preparation: Fasting for more than 12 hours, collecting venous blood in the sitting position at 8:00 am to 9:00 am.
3. Blood collection requirements: Use a vacuum blood tube with separation gel (usually yellow cap) to collect 6 ml of venous blood; avoid the occurrence of hemolysis and lipemia (If any, please specify).
4. Serum separation: 30 minutes after the collection of specimen, the specimen should centrifuge at the speed of 3000 rpm for 5 minutes within 2 hours. Divide supernatant into two collection tube without any additives, each tube should not less than 800μl. One tube sent to the central laboratory for detection, and storage the other tube as reserved sample. Attach the same number as registration form (Table 1), including name, gender, age, specimen collection time and collection unit.
5. Transportation and preservation of serum samples: serum frozen specimens should be placed in a low temperature refrigerator at -20 °C, the maximum of retention period is 6 months. Serum samples should be placed in a dry ice box while transportation. The refrigerator temperature should be recorded daily for future reference.

Table 1 Reserved sample record table and label manuscript

| Random No. | Patient name | Sex | Age | Blood collection date |
| --- | --- | --- | --- | --- |
|  |  |  |  |  |
| Hospital (detection of NT-proBNP in Qiliqiangxin study) | | | |  |

**5．Transportation**

After the researcher completes the tasks in the center (if the enrollment time is too long, within 2 months after the first patient was selected), dial the XXX express company's unified free order number: 400-0000-0000 by the account number: 0000000 for pick-up.

Please check and prepare the samples for the specific pickup address, contact person, contact phone number, delivery address, etc. The courier staff will arrive at the hospital, pack and hand over the samples.

The researcher fills in the blood sample specimen transfer order and attaches a copy of the sample record of the transport sample to the courier (the original is returned to the researcher by the auditor for the next inspection). The numbers of specimens, sample records, and transfer orders should match. The courier company should deliver the sample to the central lab within 24 hours.

**6．Receive**

The central laboratory personnel should check the blood samples, input mark code and storage the blood samples on the day the samples received. At the same time, the status of the sample should be recorded immediately.

**7．Central laboratory**

The central laboratory should complete the detection within 10 working days after receiving the sample; and at the same time, feedback the results to the leader unit and the monitor. The monitor should feedback the results to the corresponding research center within one week of receiving the test results.

Contact man: HAN Shuolong, Medical Department of Yiling Pharmaceutical

Tel: 13582167153

Email: hanshuolong@126.com

**8. Conversion between different units**

1 pg/ml = 0.118 pmol/L

pg (picogram), pmol (picomolar)

# Appendix 3: Endpoint event report process

**Researcher** Report relevant evidences and materials (medical records, laboratory results, etc) within 7 days in CRF

**CEC Secretary**  (Email) *****@***.com

Complete evidence

**Member**  (Email) Two members were appointed in turn for judgment, and the conclusion was recorded in the report.

Lack of Reserve the signed report,

evidence sent to the study secretary by email

**CEC secretary**

Inconsistency Consistency, and reply to the reporter

Save and registration

Review Researcher Record events or report SAE based on the conclusion
