# Supplementary material for: Study protocol for a randomized controlled trial: Qiliqiangxin in heart failUre: assESsment of reduction in morTality (QUEST)
Source: BMC Complement Med Ther. 2020 Feb 5;20:38. doi: 10.1186/s12906-020-2821-0 (PMC7076750; doi:10.1186/s12906-020-2821-0)
Supplement: Supplementary file 3 — Additional file 3. Informed consent form. [file 12906_2020_2821_MOESM3_ESM.zip › Appendix 3 - Informed Consent Form (ENG)R4.docx]

**Informed Consent Form**

Version No.: QUEST--ZQTY1.2

Version Date: 2018/11/02

Dear Sir or Madam:

You have been diagnosed with chronic heart failure. Therefore, we invite you to participate in a research project of the National Key R&D Program undertaken by the First Affiliated Hospital with Nanjing Medical University - “Qiliqiangxin in Heart FailUre: AssESsment of Reduction in MorTality”. The study is a post-marketing evidence-based medical study of Qiliqiangxin Capsule in the treatment of chronic heart failure, in the purpose of evaluating of Qiliqiangxin Capsule on the composite end point event of chronic heart failure. The research plan has been reviewed by the ethics committee of the First Affiliated Hospital of Nanjing Medical University, and it is considered to be in compliance with the Helsinki Declaration and in line with medical Ethic approval number: 2018-SR-275. Solution number: SP-YFC-05-QUEST. Production number of Qiliqiangxin Capsule: National Medicine Zhunzi Z20040141.

Please read the following contents carefully as much as possible before deciding whether to participate in this study. It can help you understand the research and why it is going to be conducted, the procedures and duration of the study, the benefits, risks and discomforts that may be brought to you after participating in the study. If you prefer, you can also discuss it with your relatives or friends, or ask your doctor for an explanation to help you make a decision.

1. Research background and purpose

Cardiovascular disease ranks in the top three among the global causes of death, jeopardizing human life and health. Chronic heart failure (CHF) is a series of clinical syndromes due to pump failure, reduced ejection fraction, and circulatory congestion based on a series of heart disease causing various levels of neurohumoral changes. As a end-stage of various heart disease, heart failure is becoming the most important cardiovascular disease of this century. Epidemiological data show that the number of global heart failure patients has reached 22.5 million, and the 5-year survival rate is similar to malignant tumors. With the development of social economy, the epidemiological characteristics of heart failure in developing countries are becoming more and more similar with those of developed countries. For example, coronary heart disease as the cause of heart failure is more and more prominent in China. Globally, the 1-year mortality rate of patients with heart failure over 70 years old in the United States is significantly higher than that of patients under 70 years old (22%: 13.7%). The 1-year and 3-year mortality rates of heart failure patients in Japan are 11.3% and 29.2%, respectively. In Europe, the 4-year survival rate is only 50%, and 40% of patients admitted to hospital due to heart failure may be re-admitted or die within 1 year. These means chronic heart failure is still a major threat to human life and quality of life and needs to be further investigate in this century.

Traditional Chinese medicine has accumulated practicing experience of preventing and treating heart failure, and its understanding of heart failure is also deepening with modern medicine. Qiliqiangxin Capsule has achieved significant results in the treatment of chronic left heart failure. The results of the study were published in the international journal - JACC in 2013 and received the attention and praise of international scholars. All enrollees were treated with standard-optimized heart failure treatment. Compared to placebo, Qiliqiangxin group showed a significant decrease in the NT-proBNP. It marks a major step forward for Chinese medicine to become a recognized treatment for heart failure. Qiliqiangxin Capsule became the first Chinese patent medicine to be included in the Chinese Medical Association Cardiovascular Branch " 2014 Chinese Heart Failure Guideline of Diagnosis and Treatment".

Cardiovascular mortality and heart failure recurrence rate as the primary endpoint, the purpose of this study is to further assess the clinical efficacy and safety of long-term use of Qiliqiangxin capsule, and also to investigate the efficacy characteristics and suitable population which could provide optimized treatment.

The First Affiliated Hospital of Nanjing Medical University as the leading unit, this study will conduct in over 100 hospitals in China and plans to enrolled 3,080 participants.

1. Am I suitable to participate in the study?

If you were with a clear diagnosis of chronic heart failure and have received standardized heart failure treatment for at least two weeks, you could be enrolled in the study in the study. However, if you plan to undergo cardiac resynchronization or revascularization, prepare for pregnancy, and participate in other clinical studies within one month, please inform the doctor so it would not affect your treatment. The specific inclusion criteria will be evaluated by the physician.

1. What do I need to do if I participated in the study?

The study you are attending will last for about 36 months, depending on when you enter the trial it may end early.

Before enrolling in this study, doctors will ask you a series of questions regarding you condition: ①regarding your medical history and performing a physical examination; ② Auxiliary examination included serum NT-proBNP, complete blood count, urine routine, biochemical examination, electrocardiogram, cardiac ultrasound, etc.

After the evaluation, if you meet the inclusion criteria, you will be assigned to the study group or placebo group.

Study group: Standardized treatment of chronic heart failure + Qiliqiangxin Capsules

Placebo group: Standardized treatment of chronic heart failure + Placebo Capsules

The random distribution ratio is 1:1 between study group and control group. Both groups were based on current standardized treatments for heart failure. Placebo does not contain any active ingredients, with the basis of standardized treatment for heart failure, it will not affect your current treatments. The research drugs were provided by Shijiazhuang Yiling Pharmaceutical Co., Ltd. (Approval No.: Z20040141). You will need to take the study drug in total of 12 to 36 months.

You will need to visit the hospital in the first month of enrollment, and return to the hospital every 3 months. During the visit, you will be required to bring back the remaining drugs and truthfully report the symptoms and any changes to the doctors. The doctors will collect your status and results.

In addition, it is important to tell the doctor the adjustment of any drug regimen during the study. You may not use other Traditional Chinese Medicines for chronic heart failure during the study. If you need other treatments, please contact your doctor in advanced for further evaluation. If you have any discomfort or reaction, do not hesitate to contact us.

If any serious problems (such as allergic reactions, edema, difficulty breathing, severe rash, liver and kidney damage, or heart rhythm changes) occur, you may be asked to return to the hospital for further testing and assessment.

It is not clear whether the study will affect the development of the fetus. You cannot participate in this study if you are pregnant, and you also should not breastfeed your child throughout the study. If during this time you or your partner become pregnant, the research doctor will ask you and the condition of the fetus and you will not be able to continue the study.

Please come to the hospital according to the according visit time, which is very important for this study and for the doctors to judge whether the treatment you receive really works.

1. Possible benefits of participating in the study

(1) Your condition might improve due to your participation in the study;

(2) The information obtained from this study will help the Qiliqiangxin capsule to know the efficacy and safety of the QLQX, which will be extremely useful and beneficial for patients with chronic heart failure.

1. Possible risks and adverse reactions

Please tell your family or close friend that you are participating in a clinical study and they can take note of the events described. If you have questions about your participation in the study, you can contact your doctor and medical staff.

Adverse reactions: Adverse reactions to test drugs are not known. The National Adverse Reaction Monitoring Database showed the possible reactions might included gastric discomfort such as nausea, bloating, and hiccups, etc. It is recommended to take it after meals which could improve the above symptoms.

Other risks: Although there are no allergic incident have reported, it may cause an allergic reaction in some patients as well. Other common risks included allergic at the contact of the electrocardiogram lead, bruising and/or irritating of the blood-drawing puncture site.

There may be other unknown adverse reactions. Doctors and pharmaceutical manufacturers (Shijiazhuang Yiling Pharmaceutical Co., Ltd.) will do their utmost to prevent possible adverse drug reactions during the study. Drug manufacturers will provide treatment costs and corresponding financial compensation for subjects with research-related damage or death. Shijiazhuang Yiling Pharmaceutical Co., Ltd. provides legal and economic guarantees to researchers (except those caused by medical malpractice).

1. Researcher responsibility

If your doctors aware of new information about the study drug, which may affect your decision to continue participating in the study. Doctors are obliged to inform you of this information.

If serious adverse events (such as allergic reactions, edema, difficulty breathing, severe rash, liver and kidney damage, or heart rhythm changes) occur, please communicating with your doctor. You may need to discontinue the study drug and visit to the hospital for further testing.

1. Relevant costs

This study drug (or placebo) will provide by the us freely. Also, the required testing of serum NT-proBNP, complete blood count, urine routine, biochemical examination, electrocardiogram and echocardiography will also performed by us freely.

Other treatments and tests of your standardized heart failure treatment and other preexisting diseases are not included in the study free range.

1. Information confidentiality

Your medical records (research medical records / CRFs, test orders, etc.) will be kept in the hospital you are visiting completely. The doctor will record the results of the test on your medical record. Researchers, ethics committees, and drug regulatory authorities will be allowed to access your medical records. Any public report about the results of this study will not disclose your personal identity. We will make every effort to protect the privacy of your medical information to the extent permitted by law.

1. How can I get more information?

You can ask any questions about this research at any time. The doctor will leave you the contact information. You also need to leave your contact, so that we can update any new information in time.

1. Voluntary choice to participate in research and drop out of research

Participation of this study is entirely up to your willingness. You may refuse to participate in the study or withdraw from the study at any time during the study, which will not affect your relationship with the doctor and will not affect your medical decision or personal interest. Your doctor may discontinue your participation in this study at any time for your best interests as well.

If you withdraw from the study for any reason, you may be asked about the usage condition of the test drug. You may also be asked to have laboratory and physical examinations if your doctor thinks it is needed. In order to be able to assess the safety and efficacy of the test drug, we hope that you will be able to complete the entire research process. We are looking forward to your continued participation in the study. If you decide to stop using the test drug for any reason, we will still look forward to your continued visit as planned. If you are not willing to participate in the program visit, we will follow you by phone. If you prefer, you have the option to re-start the study product and follow-up again.

If we lose contact of you, we will continue to try to get in touch with you (including not limited to your other doctors, relatives, etc.) and confirm that you continue to participate in the study to ensure your health.

1. Conclusion

It is up to you to decide whether to participate in this study. You can make a decision before discussing it with your family or friends.

Before you make a decision to participate in the study, please ask the doctor as much as possible until you fully understand the study. Thank you for reading the above materials. If you decide to participate in this study, please tell your doctor and the doctor will arrange all the research for you.

Please keep this inform consent form.

**Informed Consent Form**

**Title:** Qiliqiangxin in Heart FailUre: AssESsment of Reduction in MorTality

**Research Unit:** The First Affiliated Hospital with Nanjing Medical University

**Version No.:** QUEST--ZQTY1.2

Agreed Statement

I have read the above introduction to this study and have the opportunity to discuss and ask questions with the doctor about this study. All the questions I raised were answered.

I understand the potential benefits and risks of participating in this study. The participation of this study is fully voluntary. I confirm that I have enough time to consider this and understand that:

1. I can ask the doctor for more information at any time.
2. I can withdraw from the study at any time without discrimination and retaliation, and the medical treatment and personal interest will not be affected by that.
3. I also know that if I withdraw from the study, especially because of the study product, the change of the condition, corresponding physical examination and accessory examination will be very beneficial to me and the whole study.
4. If any other medical treatment is needed due to changes in the condition, I will seek the advice of the doctor and report the/or the researcher.
5. I agree with the drug regulatory authority, the ethics committee or the sponsor representative to consult my research materials.

I decided to participate in this study and I will follow the doctor's advice.

Subject signature：

Legal representative signature： Relationship with the subject

Tel： Date：

ID no.：

I confirm that I have explained to the subject the details of the trial, including its powers and possible benefits and risks, and gave him a copy of the signed informed consent.

Physician signature：

Tel： Date：

Principal research unit:

The First Affiliated Hospital with Nanjing Medical University (Jiangsu Provincial People's Hospital)

Ethics Committee Tel: (86) 25-68306360

（Content and interpretation of the Chinese version shall prevail）
