# Supplementary material for: Study protocol for a randomized controlled trial: Qiliqiangxin in heart failUre: assESsment of reduction in morTality (QUEST)
Source: BMC Complement Med Ther. 2020 Feb 5;20:38. doi: 10.1186/s12906-020-2821-0 (PMC7076750; doi:10.1186/s12906-020-2821-0)
Supplement: Supplementary file 3 — Additional file 3. Informed consent form. [file 12906_2020_2821_MOESM3_ESM.zip › Appendix 3 - Informed Consent Form (CN)R4.doc]

**知情同意书·知情告知页**

版本号：QUEST--ZQTY1.2

版本日期:2018年11月2日

亲爱的受试者：

您已被确诊患有慢性心力衰竭，我们邀请您参加一项由南京医科大学第一附属医院承担的国家重点研发计划专项课题研究，研究为芪苈强心胶囊治疗慢性心衰的上市后的循证医学研究，本研究名称为“芪苈强心胶囊对慢性心衰复合终点事件的评估研究”，研究方案已经得到组长单位南京医科大学第一附属医院伦理委员会审核，认为是遵从赫尔辛基宣言，符合医学伦理道德的，同意进行临床研究，伦理批号：2018-SR-275。方案编号：SP-YFC-05-QUEST。芪苈强心胶囊生产批准文号为：国药准字Z20040141。

在您决定是否参加这项研究之前，请尽可能仔细阅读以下内容。它可以帮助您了解该项研究以及为何要进行这项研究，研究的程序和期限，参加研究后可能给您带来的益处、风险和不适。如果您愿意，您也可以和您的亲属、朋友一起讨论，或者请医生给予解释，帮助您做出决定。

1. **研究背景和研究目的**

在全球范围的死亡原因中心血管疾病位列前三，严重地危害着人类的生命和健康。慢性心力衰竭（CHF）是在器质性心脏病基础上发生泵功能衰竭、射血分数减少、循环淤血，同时出现一系列神经体液改变的一系列临床综合症。心衰作为各种心脏病发展的严重阶段，正在成为本世纪最重要的心血管病症。有流行病学资料显示，目前全球心衰患者的数量已高达2250万，且5年存活率与恶性肿瘤相仿。随着流行病学的变迁和社会经济的发展，发展中国家心衰的流行病学特点与发达国家日益相近，如冠心病作为心衰的病因在我国显得越来越突出。美国70岁以上的心衰患者1年死亡率明显较70岁以下患者高（22％：13.7％）。日本的心衰患者1年和3年死亡率分别为11.3％和29.2％，在欧洲，4年生存率仅为50％，而且有40％因心衰入院的患者将可能在1年内再次入院治疗或者死亡。慢性心衰依然是严重威胁人类生命和生活质量的主要问题，所以国际上仍将慢性心衰作为本世纪需要解决的重要课题。

中医学在防治心力衰竭的长期医疗实践中，积累了丰富的经验，对心衰的认识也在不断深化。芪苈强心胶囊治疗慢性左心衰竭取得重大临床研究成果，该研究结果于2013年发表于国际心血管病权威杂志《JACC》上，并受到国外学者的关注和好评。所有入选者均采用了标准优化治疗，加用芪苈强心胶囊组较安慰剂组，氨基末端端利钠肽前体（NT-proBNP）显著降低，且降幅＞30%的患者比率也显著增加，标志着中药成为心衰公认治疗药物向前迈进一大步。芪苈强心胶囊成为第一个列入中华医学会心血管病学分会《中国心力衰竭诊断和治疗指南2014》的中成药。

本项研究目的以心血管死亡率与心衰复发率为主要研究终点，进一步阐明芪苈强心胶囊长期用药的临床疗效及安全性，明确疗效特点及适宜人群，为优化临床合理用药方案提供高质量临床证据。

本研究将以南京医科大学第一附属医院为组长单位的近100家医院进行研究，计划有3080例受试者自愿参加。

1. **参加研究的适合人群和不适合的人群**

明确诊断患有慢性心衰的受试者，经过医生诊断您符合该研究的入选、排除条件可以参加本研究。但如果您计划于近期内进行心脏再同步化或血运重建、准备妊娠、1个月内参加过其他临床研究等情况请如实告知医生，以免影响医生对您的治疗。

1. **如果参加研究将需要做什么？**

您参加的此项研究将持续约36个月，但是也可能提早结束，这取决于您进入试验的时间。

在入选本项研究前，您将接受以下检查以确定是否可以参加研究：①医生将询问、记录病史、并进行体格检查。②进行血清NT-proBNP检查，血常规、尿常规、生化检查、心电图、心脏超声等理化检查。

检查结束后，如果您符合入选条件，您将被分入到试验组或安慰剂组，试验组为心衰标准治疗+芪苈强心胶囊，安慰剂组为心衰标准治疗+芪苈强心胶囊模拟剂。受试者分配入两组的机会均等各为50%。两组均在目前心衰标准化治疗基础上进行，安慰剂组中安慰剂不含有任何有效成分而且是在心衰标准化治疗基础使用，不会影响您目前心衰治疗药物的使用。研究药物均由石家庄以岭药业股份有限公司提供（批准文号：Z20040141）。服用方法为每次4粒，每日3次。共服用12-36个月时间，您需要按照访视计划于入组第1个月，第3个月此后每3个月到医院复诊，带回所剩余的药物，并如实向医生反映病情变化，医生将收集病史、体检结果。在研究期间您不能使用治疗慢性心衰的其它中药，如您需要进行其它治疗，请事先与您的医生取得联系。如果您有任何不适或反应，请及时通知医师，以便及时得到处理。

如果发生严重问题（比如过敏反应、浮肿、呼吸困难、严重皮疹、肝肾损伤或心律改变），可能会要求您回到医院进行进一步的检查，包括更多的血液学检查。

如果您同时服用研究药物和常规研究药物或辅助药物将改变药物的作用方式。研究期间请告诉医生您正在服用哪些药物非常重要。

如果您在妊娠期则不能参加本研究。在整个研究期间，作为母亲，您也不能母乳喂养孩子。

在研究期间您不能怀孕或使伴侣怀孕，因为现在尚不明确该研究药物是否会影响胎儿的发育。

请您按医生和您约定的访视时间来医院就诊，这对于本项研究非常重要，因为医生将判断您所接受的治疗是否真正起到作用。

1. **参加研究可能的受益**
（1）您因参加本研究病情可能得到改善；

（2）从本研究中得到的信息将有助于芪苈强心胶囊获得该药治疗慢性心衰患者的有效性和安全性资料。这些信息将有益于帮助将来的患者。

1. **参加研究可能的不良反应、风险**

您需告诉您的家人或与您亲近的朋友您正在参加一项临床研究，他们可以注意上面描述的事件。如果他们对您参加研究有疑问，您可以告诉他们怎样联系您的研究医生。

不良反应：试验药物不良反应尚不明确。国家不良反应监测数据库检索可见恶心、腹胀、呃逆等胃部不适，建议饭后服用可改善上述症状。

其他风险：

心电图检查时，在监测设备接触处可能会出现皮肤发痒。

抽取血样时，您可能会感觉乏力或者轻微的疼痛、或者抽取部位瘀青、刺激或发红。也可能会发生感染，但较罕见。

也许还有其他未知的不良反应出现。比如，所有的药物在某些患者中都可能引起过敏反应。

医生和药品生产企业（石家庄以岭药业股份有限公司）将尽全力预防由于本研究可能出现的药物不良反应。药品生产企业将对与研究相关的损害或死亡的受试者提供治疗费用及相应的经济补偿。石家庄以岭药业股份有限公司向研究者提供法律上与经济上的担保，但由医疗事故所致者除外。

1. **研究者职责**

如果您的医生得知一些关于研究药物的新信息，这些信息可能影响您继续参加研究的决定，医生会告知您这些信息。

如果发生严重问题（比如过敏反应、浮肿、呼吸困难、严重皮疹、肝肾损伤或心律改变），可能会要求您回到医院进行进一步的检查，包括更多的血液学检查。如有需要您的医生将向您解释这些检查。与您的医生沟通后也许您需要停用研究药物。

如果在此期间您或您的伴侣怀孕了，研究医生将询问您及胎儿的状况，您将无法继续参加研究。

1. **有关费用**

此项研究为常规指南治疗基础上加用试验药物，不会额外增加您的经济负担，同时研究开始和过程中医生将安排您进行血清NT-proBNP检查，血常规、尿常规、生化检查、心电图、心脏超声检查均为免费。研究期间的研究药物也是免费的。因此将不提供其他补偿（包括交通补偿）。

对于您同时合并的治疗心衰和其他疾病所需的治疗药物和检查，也不在免费的范围之内。

1. **个人信息是保密的吗？**

您的医疗记录（研究病历/CRF、化验单等）将完整地保存在您所就诊的医院。医生会将化验检查结果记录在您的病历上。研究者、伦理委员会和药品监督管理部门将被允许查阅您的医疗记录。任何有关本项研究结果的公开报告将不会披露您的个人身份。我们将在法律允许的范围内，尽一切努力保护您个人医疗资料的隐私。

1. **怎样获得更多的信息？**

您可以在任何时间提出有关本项研究的任何问题，并得到相应的解答。医生将给您留下医院和自己的联系方式以便能回答您的问题。您也需要留下自己的联系方式，在研究过程中有任何重要的新信息，可能影响您继续参加研究的意愿时，您的医生将会及时通知您。

1. **可以自愿选择参加研究和中途退出研究**

是否参加本项研究完全取决于您的自愿。您可以拒绝参加此项研究，或在研究过程中的任何时间退出研究，这都不会影响您和医生的关系，都不会影响对您的医疗或有其他方面利益的损失。

医生出于对您的最大利益考虑，可能会随时中止您参加本项研究。

如果您因为任何原因从研究中退出，您可能被询问有关使用试验药物情况。如果医生认为需要，您也可能被要求进行实验室检查和体格检查。

如果您选择参加本项研究，我们希望您能够坚持完成全部研究过程。为了能够评估试验药物的安全性和有效性，我们期待您坚持参加研究直至研究结束。如果您由于任何原因决定停止使用试验药物，我们仍期待您继续按计划参加访视。如果您不愿意参加计划访视，我们将对您进行电话随访。如果您愿意，您可选择选择重新开始研究。

如果您失去联系，我们将持续尝试与您取得联系（包括不限于您的其他医生，亲属等）并确认您是否继续参加研究，以确保您的健康状况。

1. **现在该做什么？**

是否参加本项研究由您自己决定。您可以和您家人或者亲友讨论后再做出决定。

在您做出参加研究的决定前，请尽可能向医生询问有关问题，直至您对本项研究完全理解。

感谢您阅读以上材料。如果您决定参加本项研究，请告诉医生，医生会为您安排一切有关研究的事务。

请您保留这份资料。

**知情同意书·同意签字页**

**项目名称：**芪苈强心胶囊对慢性心衰复合终点事件的评估研究

**研究单位:** 南京医科大学第一附属医院

**版本号：**QUEST--ZQTY1.2

**同意声明**

1. 我已阅读了上述有关本研究的介绍，而且有机会就此研究与医生讨论并提出问题。我提出的所有问题都得到了满意的答复。

2. 我知道参加本研究的受益和风险。我知道参加研究是自愿的，我确认已有充足的时间对此进行考虑，而且明白：

（1）我可以随时向医生咨询更多的信息；

（2）我可以随时退出本研究，而不会受到歧视和报复，医疗待遇与权益不会受到影响；

（3）我同样清楚，如果我中途退出研究，特别是由于药物的原因使我退出研究时，我若将病情变化告诉医生，完成相应的体检和理化检查，这将对我和整个研究十分有利；

（4）如果因病情变化需要采取任何其他的药物治疗，我会在事先征求医生的意见，或在事后如实告诉医生；

（5）我同意药品监督管理部门，伦理委员会或申办者代表查阅我的研究资料；

最后，我决定同意参加本项研究，并保证尽量遵从医嘱。

受试者签名：	（或）法定代理人签名：	与受试者关系

联系电话：	年	月	日

受试者身份证号码：

我确认已向受试者解释了本试验的详细情况，包括其权力以及可能的受益和风险，并给其一份签署过的知情同意书副本。

医生签名：

联系电话：	年	月	日

组长单位南京医科大学第一附属医院（江苏省人民医院）伦理委员会联系电话：025-68306360
